# Achievement of Guideline-Recommended Targets for Secondary Prevention of Cardiovascular Disease in 38 Low-Income and Middle-Income Countries

**DOI:** 10.1007/s44197-024-00251-3

**Published:** 2024-06-03

**Authors:** Zhiguang Liu, Minghai Yan, Lap Ah Tse, Yingxuan Zhu, Xinyue Lang, Xin Liu, Yang Lin, Bo Hu

**Affiliations:** 1 Medical Research and Biometrics Center, National Clinical Research Center for Cardiovascular Diseases, Fuwai Hospital, National Center for Cardiovascular Diseases, Peking Union Medical College & Chinese Academy of Medical Sciences, Beijing, China; 2 Clinical Trial Unit, Beijing Anzhen Hospital, Capital Medical University, Beijing, China; 3 JC School of Public Health and Primary Care, The Chinese University of Hong Kong, Hong Kong SAR, China

**Keywords:** Cardiovascular disease, Low-income and middle-income countries, Secondary prevention, Global health

## Abstract

**Background:**

This study aimed to estimate the prevalence of achieving the secondary prevention targets recommended in the World Health Organization (WHO) guidelines for cardiovascular disease (CVD) in 38 low-income and middle-income countries (LMICs).

**Methods:**

We pooled nationally representative cross-sectional surveys from 38 LMICs between 2013 and 2020. Treatment, metabolic and lifestyle targets were assessed for individuals with a self-reported history of CVD according to WHO’s recommendations. Associations between the prevalence of guideline adherence and sociodemographic characteristics were assessed using multivariate Poisson regression models.

**Results:**

The pooled sample included 126 106 participants, of whom 9821 (6.8% [95% CI 6.4–7.2]) reported a history of CVD. Overall, the prevalence of achieving treatment targets in patients with CVD was 22.7% (95% CI, 21.0-24.5%) for antihypertensive drugs, 19.6% (17.9-21.4%) for aspirin, and 13.6% (12.0-15.44%) for statins. The prevalence of achieving metabolic targets was 54.9% (52.5-57.3%) for BMI, 39.9% (37.7-42.2%) for blood pressure, 46.1% (43.6-48.6%) for total cholesterol, and 84.9% (83.1-86.5%) for fasting blood glucose. The prevalence of achieving lifestyle targets was 83.2% (81.5-84.7%) for not smoking, 83.1% (81.2-84.9%) for not drinking, 65.5% (63.1-67.7%) for sufficient physical activity and 16.2% (14.5-18.0%) for healthy diet. Only 6.1% (5.1-7.4%) achieved three treatment targets, 16.0% (14.3-17.9%) achieved four metabolic targets, and 6.9% (5.8-8.0%) achieved four lifestyle targets. Upper-middle income countries were better than low-income countries at achieving the treatment, non-drinking and dietary targets. Being younger and female were associated with poorer achievement of metabolic targets.

**Conclusion:**

In LMICs, achieving the targets recommended in the guideline for treatment, metabolism and healthy lifestyles for patients with CVD is notably low. This highlights an urgent need for effective, systematic secondary prevention strategies to improve CVD management.

**Supplementary Information:**

The online version contains supplementary material available at 10.1007/s44197-024-00251-3.

## Introduction

The burden of cardiovascular disease (CVD) and mortality in low-income and middle-income countries (LMICs) has been increasing for decades. In 2019, 4.96 million deaths were caused by CVD in LMICs [1]. However, more than 40% of CVD cases occur annually in people with a history of CVD [2]. Risk factor reduction and the adoption of evidence-based interventions are the cornerstones of preventing CVD recurrence and mortality [3].

Adherence to treatment and targets for metabolic and behavioural risk factors are important components of quality CVD management [4]. Several guidelines have emphasised that secondary prevention of CVD should focus on triple therapy with aspirin, statins and antihypertensives, control of metabolic risk factors such as body mass index (BMI), blood pressure, blood glucose and cholesterol; and adoption of a healthy lifestyle with smoking cessation, alcohol restriction, rational diet and sufficient physical activity [5, 6]. However, adherence to guidelines is poorer in populations with a history of CVD, particularly those living in LMICs. The Prospective Urban Rural Epidemiology (PURE) study, using data on patients with CVD collected in 17 LMICs before 2009, found that the use of antiplatelet drugs was 25.3%, statins 14.6%, β blockers 17.4% and only 4.3% adhered to a healthy lifestyle of smoking cessation, a healthy diet and sufficient physical activity [7, 8]. The most recent data, 10 years later, show that aspirin and statins use in LMICs has increased to 28.5% and 21.9% respectively, but still falls short of the guidelines [9–11]. However, these studies ignored the young age structure of LMICs and excluded CVD patients younger than 40 years from the analysis framework. In fact, there is a global trend of younger onset of CVD, and premature CVD has higher recurrence and mortality [12, 13].

Studies in several high-income countries have shown low prevalence of guideline adherence for metabolic and behavioural risk factors, but high prevalence of medication adherence in adults with established CVD [14–16]. However, medication, metabolic and lifestyle adherence among patients with CVD in LMICs is unknown, posing a major challenge for secondary prevention of CVD. The aim of this study was to estimate the prevalence of achieving the secondary prevention targets among patients aged 18–69 with CVD in 38 LMICs, using data from a nationally representative cross-sectional survey, and to assess its association with various sociodemographic characteristics.

## Methods

### Study Design and Data Source

We pooled and analysed individual participant data from nationally representative cross-sectional surveys conducted by WHO in 38 LMICs between 2013 and 2020. The data were obtained from the Stepwise Approach to Surveillance (STEPS) survey, a community-based study initiative thoroughly described in previously published literature [17].

The sample consisted of participants aged 18–69 years. We chose this age range not only to be consistent with the WHO guidelines for CVD management and the age range recommended for surveillance in Sustainable Development Goal (SDG) 3.4 [18, 19], but also to account for the unique age structure of LMICs and the trend towards younger onset of CVD. In addition, the upper age limit for most national surveys was set at 69 years.

### Outcomes and Procedures

The primary outcomes are the prevalence of people with a self-reported history of CVD who achieved the targets recommended by the WHO CVD management guidelines for treatment (antihypertensive medication, aspirin and statins), metabolism (BMI, blood pressure, blood glucose, and total cholesterol), and healthy lifestyle (smoking cessation, alcohol restriction, rational diet, and sufficient physical activity). In the STEPS study, a history of CVD (heart attack or stroke) was obtained from each participant using a standardised questionnaire, i.e. “Have you ever had a heart attack or chest pain from heart disease (angina), or a stroke (cerebrovascular accident or incident)?”.

Self-reported use of antihypertensive medication, aspirin, and statins were the treatment outcomes of interest. Aspirin and statin use was assessed in individuals with a history of CVD by the questionnaire question “Are you currently taking aspirin/ statins regularly to prevent or treat heart disease?”. The use of antihypertensive medication was assessed in individuals with self-reported hypertension by the question “In the past two weeks, have you taken any medication for raised blood pressure prescribed by a doctor or other health worker?”.

Weight and height were measured by trained staff, and BMI was calculated as weight divided by the square of height. After participants had rested and stabilised on site, staff took three or two measurements of blood pressure and used the mean of the measurements to determine systolic and diastolic blood pressure. After performing an overnight fast, venous blood samples were taken to measure fasting blood glucose and total cholesterol. The BMI target was set at < 25 kg/m². For South East Asia, the BMI threshold was set at < 23 kg/m². The blood pressure target was set at < 130/80 mm Hg. The target for fasting blood glucose was set at < 6.1 mmol/L (110 mg/dL). The total cholesterol target was set at < 4.0 mmol/L (152 mg/dL).

Healthy lifestyle status (smoking, alcohol consumption, diet and physical activity) was assessed by self-report questionnaire. Not currently smoking was defined as not having used tobacco products in the past 12 months [8]. Not currently drinking was defined as not consuming alcohol in the past 30 days [20]. Rational diet was defined as an average of more than 5 portions of fruit and/or vegetables per day [18, 21]. Sufficient physical activity was defined as more than 150 min of moderate-intensity activity at work or leisure time, or more than 75 min of equivalent vigorous-intensity activity per week [22].

All of the above treatment, metabolic and lifestyle targets were based on the WHO guidelines for the prevention of CVD published in 2008 [18].

### Statistical Analysis

We took into account the STEPS multistage stratified whole cluster randomised sampling study design and included pre-calculated weights from the survey team. In all analyses, the Stata ‘svyset’ command was used to adjust for stratification and clustering effects of primary sampling units. To account for population error, selection error and non-response error, the weights for the pooled sample and the subsamples of interest were rescaled based on the proportion of the population aged 18–69 years in the 38 countries, treating the country level as a fixed effect.

First, we reported the prevalence of adherence to treatment (antihypertensive drugs, aspirin, and statins), metabolic (BMI, blood pressure, blood glucose, and total cholesterol), and lifestyle (not currently smoking, not currently drinking, rational diet, and sufficient physical activity) guidelines overall and by World Bank income group, respectively. Second, the prevalence of all components within the treatment, metabolic, and lifestyle groups that were met was calculated by WHO region and World Bank income group. Finally, to explore individual characteristics associated with adherence to each guideline recommendation in the pooled sample, we fitted multivariable Poisson regression models adjusted for age (18–34, 35–44, 45–54 and 55–69 years), sex, and education (no formal schooling, primary and secondary or higher), and used the average marginal effect model to estimate absolute differences in guideline achievement prevalence.

We carried out some sensitivity analyses. First, we adjusted to give equal weight to each country. Second, we estimated the prevalence of achieving the treatment target in people aged 40 years and older. Third, the blood pressure target was set at < 140/90 mm Hg and the total cholesterol target was set at < 5.0 mmol/L (190 mg/dL). Analyses were performed in Stata version 18.0 and R version 4.2.2. S1 Text provides further methodological details.

## Results

### Survey Characteristics

The study included 9821 people with CVD aged 18–69 years in 38 LMICs, with a median age of 49 years (interquartile range, 36–59 years), 6256 women (56.1% [95% CI 53.4–58.0] of the population-weighted sample) and 3565 men (43.9% [95% CI 42.0-46.6] of the population-weighted sample). The number of surveys varied by region: 2 in the Americas, 5 in South-East Asia, 6 in the Eastern Mediterranean, 6 in the Western Pacific, 8 in Europe, and 11 in Africa. In addition, 9 surveys were conducted in LIC, 18 in L-MIC, and 11 in UMIC. The median response rate for all surveys was 87% (74-95%) (Table 1; Table S2).

### Treatment, Metabolic and Lifestyle Targets for Secondary Prevention of CVD across the Pooled Sample and by Income Group

**Fig. 1:**
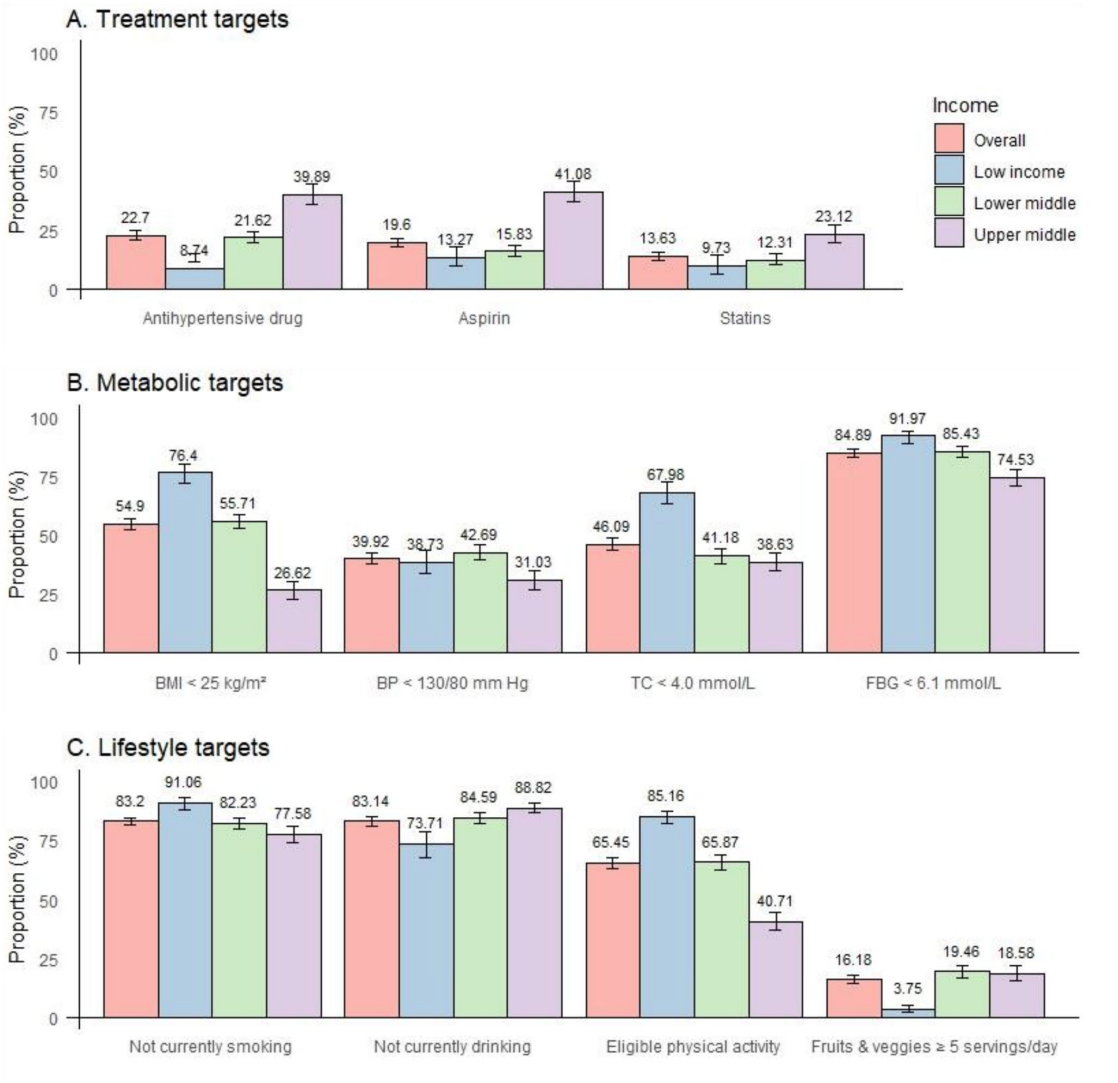
Prevalence of treatment, metabolic and lifestyle targets for secondary prevention of cardiovascular disease. Sample weights are re-adjusted based on each country’s 2019 population aged 18–69. Error bars represent 95% CIs. BMI = body mass index, BP = blood pressure, TC = total cholesterol, FBG = fasting blood-glucose. Bangladesh, Myanmar, Nepal, Sir Lanka and Timor-Leste have a BMI target of < 23 kg/m^2^

The prevalence of adults with a self-reported history of CVD meeting treatment targets for antihypertensive drug use was 22.7% (95% CI, 21.0-24.5%). At the level of country income subgroups, the prevalence was 39.9% in UMIC, 21.6% in L-MIC and 11.5% in LIC. Secondary prevention with statins was 13.6% (12.0-15.44%). 23.1% in UMIC, 12.3% in L-MIC and 9.7% in LIC. Secondary prevention with aspirin was 19.6% (17.9-21.4%). 41.1% in UMIC, 15.8% in L-MIC and 13.3% in LIC (Fig. 1; Table S3).

The prevalence of metabolic targets with a BMI below 25 kg/m² was 54.9% (95% CI, 52.5-57.3%). UMIC, L-MIC and LIC were 26.6%, 55.7% and 76.4% respectively. Blood pressure below 130/80 mm Hg was 39.9% (37.7-42.2%). 31.0% in UMIC, 42.7% in L-MIC and 38.7% in LIC. 46.1% (43.6-48.6%) had a total cholesterol below 4.0 mmol/L. 38.6% in UMIC, 41.2% in L-MIC and 68.0% in LIC. Fasting blood glucose below 6.1 mmol/L was found in 84.9% (83.1-86.5%). 74.5% in UMIC, 85.4% in L-MIC and 92.0% in LIC.

The prevalence of achieving the lifestyle target of not currently smoking was 83.2% (95% CI, 81.5-84.7%). Specifically, 77.6% in UMIC, 82.2% in L-MIC and 91.1% in LIC. The prevalence of current non-drinkers was 83.1% (81.2-84.9%). 88.8% in UMIC, 84.6% in L-MIC and 73.7% in LIC. 65.5% (63.1-67.7%) were sufficiently physically active. 40.7% in UMIC, 65.9% in L-MIC and 85.2% in LIC. 16.2% (14.5-18.0%) consumed ≥ 5 portions of fruit and vegetables per day. 18.6% in UMIC, 19.5% in L-MIC and 3.8% in LIC. Table S3 shows the prevalence of treatment, metabolic and lifestyle targets achieved in 6 WHO regions and 38 countries.

### Combination of Treatment, Metabolic and Lifestyle Targets Across the Pooled Sample, by Income Group and Region

**Fig. 2:**
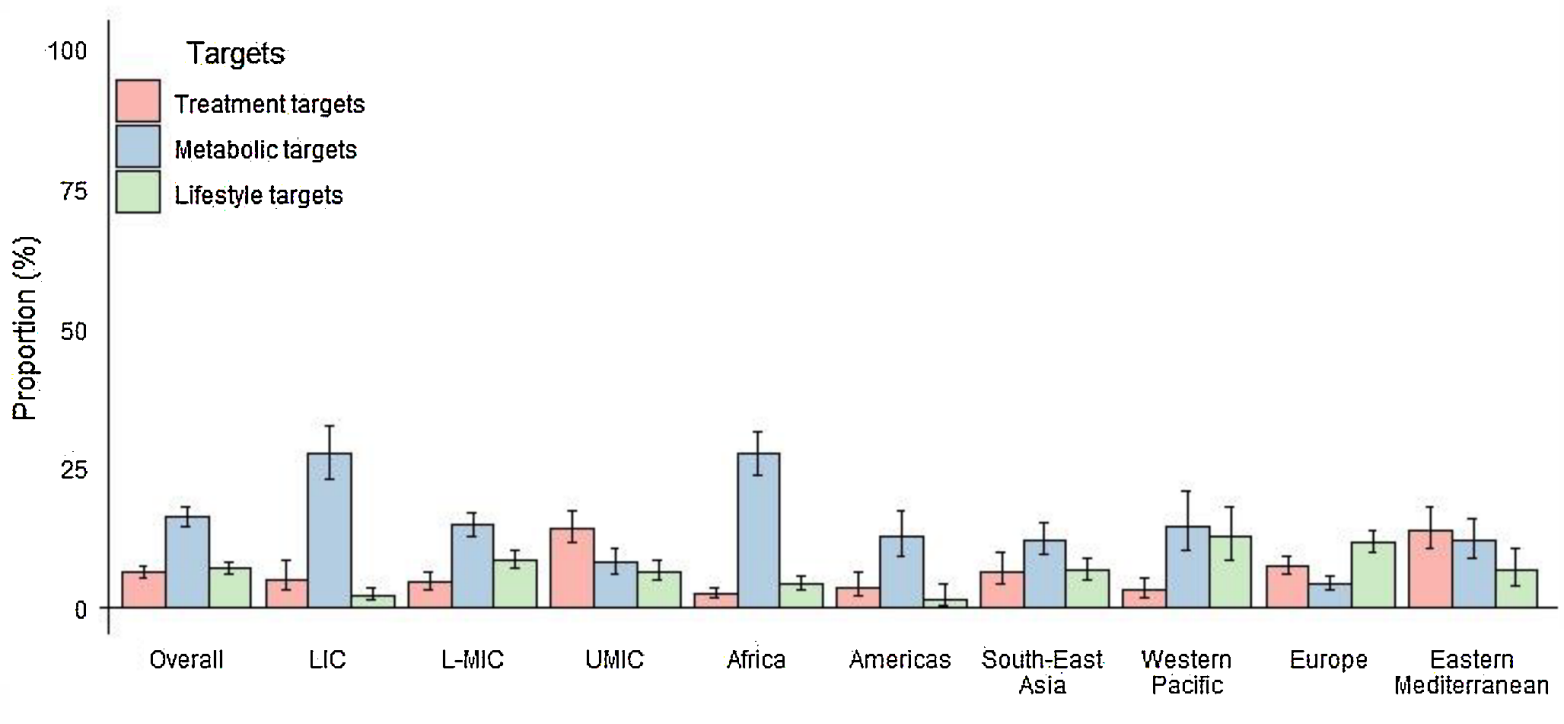
Prevalence of guideline-recommended targets for all treatment, metabolic and lifestyle components in CVD patients by World Bank income group and region. Sample weights are re-adjusted based on each country’s 2019 population aged 18–69. Error bars represent 95% CIs. LIC = low-income country, L-MIC = lower middle-income country, UMIC = upper-middle-income country. Treatment targets include antihypertensive medication, aspirin and statin use. Metabolic targets include BMI < 25 kg/m^2^, blood pressure < 130/80 mm Hg, blood glucose < 6.1 mmol/L (110 mg/dL), and total cholesterol < 4.0 mmol/L (152 mg/dL). Bangladesh, Myanmar, Nepal, Sir Lanka and Timor-Leste have a BMI target of < 23 kg/m^2^. Lifestyle targets include non-smoking, non-drinking, sufficient physical activity and rational diet

Overall, the prevalence of achieving treatment targets for concomitant use of three medications was 6.1% (95% CI, 5.1-7.4%), with the highest prevalence in the UMIC and Eastern Mediterranean regions (14.1% and 13.8%) and the lowest prevalence in the L-MIC and Western Pacific regions (4.4% and 3.0%). The prevalence of achieving all four metabolic targets simultaneously was 16.0% (14.3-17.9%). LIC and Africa had the highest prevalence at 27.5% and 27.5%, respectively. UMIC and Europe had the lowest prevalence at 7.9% and 4.0%. The prevalence of achieving four healthy lifestyle targets simultaneously was 6.9% (5.8-8.0%). L-MIC and Western Pacific had the highest prevalence at 8.5% and 12.5%. LIC and the Americas had the lowest prevalence at 2.1% and 1.2% (Fig. 2; Table S4).

### Treatment, Metabolic and Lifestyle Targets for Secondary Prevention of CVD Across Individual Characteristics

**Fig. 3:**
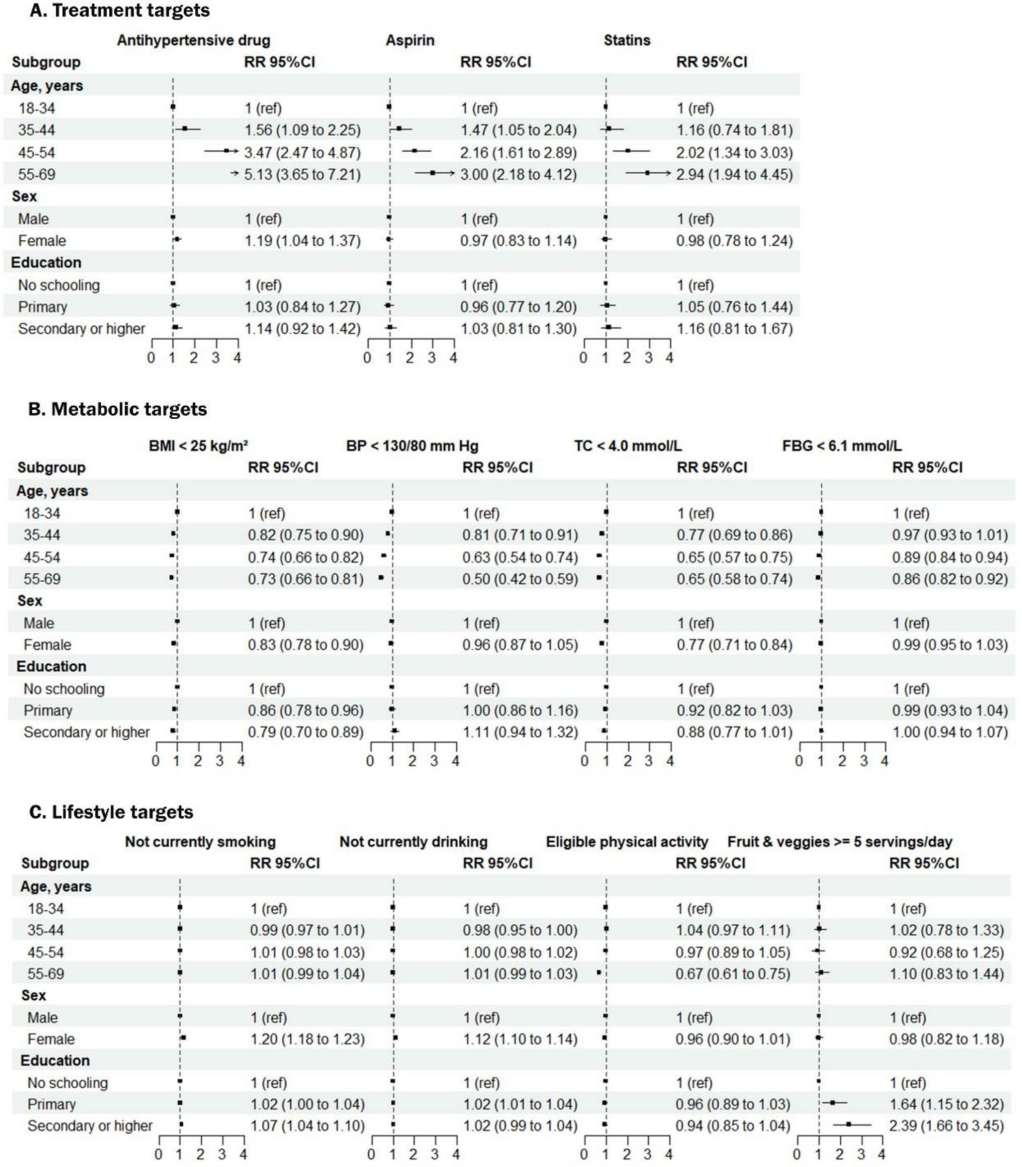
Association between individual characteristics and guideline-recommended treatment, metabolic and lifestyle targets for cardiovascular disease patients. Sample weights are re-adjusted based on each country’s 2019 population aged 18–69. Error bars represent 95% CIs. BMI = body mass index, BP = blood pressure, TC = total cholesterol, FBG = fasting blood-glucose. The models have been adjusted for age group, sex and education, with the inclusion of country-level fixed effects. Bangladesh, Myanmar, Nepal, Sir Lanka and Timor-Leste have a BMI target of < 23 kg/m^2^

In the overall pooled sample, older age was associated with a higher prevalence of achieving treatment targets for antihypertensives, statins, and aspirin, but a lower prevalence of controlling BMI, blood pressure, total cholesterol, and blood glucose. In addition, women were more likely than men to take antihypertensives for secondary prevention of CVD and to maintain a healthy lifestyle of non-smoking and non-alcohol consumption, but had poorer control of BMI and total cholesterol. In addition, higher education was associated with poorer BMI control and better dietary quality (Fig. 3; Table S5).

### Sensitivity Analysis

When all countries were equally weighted, the prevalence did not change significantly. The prevalence of drug use increased when the population was restricted to those aged 40 years and over. When the blood pressure target was 140/90 mm Hg, the achievement prevalence was 67.4% (65.4-69.4%). When the total cholesterol target was 5.0 mmol/L, the achievement prevalence was 75.5% (73.3-77.6%) Table (S6).

## Discussion

In a nationally representative cross-sectional survey conducted in 38 LMICs, the recommended targets of the CVD secondary prevention guidelines were poorly met. For treatment targets, about one-fifth of patients were taking antihypertensive drugs or aspirin, and one-seventh were taking statins. Regarding metabolic targets, only about half of the patients had a BMI of < 25 kg/m^2^ or total cholesterol of < 4.0 mmol/L, while blood pressure control rate was even worse. With the exception of diet, all lifestyle goals were all achieved in more than 60% of patients. However, fewer than one in ten patients achieved three treatment targets or four lifestyle targets, and fewer than one in three patients achieved four metabolic targets. At the national level, UMIC was better than L-MIC and LIC at achieving treatment, non-drinking and fruit and vegetable lifestyle targets, but worse than L-MIC and LIC at achieving metabolic, non-smoking and physical activity targets. At the level of individual characteristics, adherence to treatment and metabolic targets was lower in younger patients, and adherence to metabolic targets was lower in women.

The results of our study underscore once again that drugs for secondary prevention of CVD are underused in LMICs and even worse in LIC. Achievement of treatment targets was the lowest compared with metabolic and lifestyle targets. The results of the PURE study at the community level in 17 countries more than a decade ago are similar to what we observed now [7]. This suggests that the prevalence of essential medicines among CVD patients in LMICs has not been improved over a period of more than a decade. It also suggests that LMICs will struggle to meet the target of at least 50% of eligible people using therapeutic medicines by 2030, as proposed by the United Nations SDG 3.4 [23]. Our study showed that only Belarus, Iraq and Turkmenistan met the 50% target for antihypertensives; Belarus, Iraq, Jordan, Lebanon and Turkmenistan met the 50% target for aspirin; and no country met the 50% target for statins. In LMICs, drug supply shortages, coupled with a lack of adequate patient access to healthcare, have left many people without access to secondary prevention medicines [24]. In LMICs, there is a lack of financial protection against health care costs for CVD patients, and CVD households have a high incidence of catastrophic expenditure and poverty, and patients cannot afford secondary prevention medicines [25]. Moreover, we found poor medication use among younger patients. This may be due to concerns about medication side effects in younger patients, a lower perceived risk of CVD, and a lack of evidence from randomised controlled trials that could be used to guide secondary prevention medication in young people [26, 27].

Achievement of glucose guideline-recommended targets was better, but BMI, blood pressure, and lipid control were poor in patients with CVD. Achievement of four metabolic targets was better in LIC than in L-MIC and UMIC. Metabolic control was worse in the older people and in women. These results are similar to previous studies [28–30]. Differences in diet, culture, and physical activity patterns unique to each country, as well as individual access to health care services, may contribute to differences in metabolic target achievement between countries [31]. The high metabolic burden of UMIC may be driven by an ageing population [32]. Nevertheless, UMIC has the lowest CVD burden and mortality compared to L-MIC and LIC, which is associated with better control of risk factors by people living there and a high prevalence of medication adherence and use of revascularization surgery [33, 34]. In the future, greater efforts should be made to promote weight and lipid management in patients with CVD, with a focus on older patients and women, to prevent health inequalities from worsening.

Lifestyle attainment was higher for not smoking and not drinking alcohol, but lower for physical activity and diet. Achievement for physical activity was more than twice as high in LIC than in UMIC, and achievement for diet was five times higher in UMIC than in LIC. These findings are similar to those of previous studies [1, 35, 36]. Compared with the PURE study, our results showed an increase in physical activity levels and a significant decrease in the prevalence of healthy eating [8]. This may be due to the inclusion of more LMICs in our study, and the higher proportion of rural population in our data than in the PURE study (67.6% vs. 39.4%), and people in these areas may have less access to and affordability of fruits and vegetables and lower purchasing power [37]. CVD patients in LMICs or living in rural communities were more likely to be employed in manual occupations and therefore showed improved prevalence of physical activity, but physical activity levels in UMICs remain a concern. Governments in UMICs should provide more accessible opportunities for leisure activities to improve overall physical activity levels.

Overall, the prevalence of achieving three treatment targets, four metabolic targets or four healthy lifestyle targets is unacceptably low and shows national and regional variations. This may be due to a combination of factors such as economy, education, level and quality of universal health coverage, medications, and access to healthy communities. In any case, these results suggest that our efforts to manage patients with CVD are far from adequate. Some innovative approaches to secondary prevention should be implemented in LMICs. A fixed-dose combination pill can simplify treatment and improve adherence, while combining safety and cost-effectiveness, and has the potential to be an effective way to improve the global secondary prevention dilemma [38, 39]. Personalised intervention strategies based on non-physician health workers (NPHWs) are effective in improving adherence to guideline-recommended medications and healthy lifestyles in patients with CVD [40]. Cardiac rehabilitation is a guideline-recommended, cost-effective, and scalable strategy for the secondary prevention of CVD [41]. Monitoring physical activity and providing feedback and advice to help patients change unhealthy behaviours using wearable activity trackers can improve self-management in patients with CVD [42].

Our study has several limitations. First, we were unable to obtain detailed metabolic data on CVD subtypes, haemoglobin A1c and low-density lipoprotein, as recommended by current guidelines. We were also unable to obtain detailed data on types of antihypertensive medication and diet, which may have led an overestimation of the results. Second, recall bias was unavoidable due to patients’ self-reported history of CVD and medication use, among other factors. Third, slight differences in survey design between countries may have increased the heterogeneity of the pooled sample, although the weights were adjusted in our analyses. Fourth, the survey was conducted between 2013 and 2020 and may not reflect the current status of CVD secondary prevention in LMICs. Fifth, the multivariate Poisson regression model adjusted only for information on basic demographic characteristics and did not account for unmeasured confounders, which may increase the risk of false positives. In addition, the variable of living in an urban or rural community was not included in the multivariable analysis due to the high percentage of missing data, and the results must be interpreted with caution. Sixth, additional sensitivity analysis could not be performed due to lack of information on CVD subtypes. Finally, there are large differences in the level of universal health coverage and health care capacity between countries, and our results cannot be extrapolated to other LMICs and regions. The implementation of secondary prevention of CVD in more LMICs and regions should be continuously monitored in the future.

## Conclusions

In LMICs, a significant gap exists between the achievement of guideline-recommended secondary prevention in CVD patients and the ideal state. The prevalence of meeting medication, BMI, blood pressure, total cholesterol, physical activity, and dietary targets is extremely low, with very few participants achieving all combined targets. It is crucial to develop CVD secondary prevention strategies that are effective, affordable, and tailored to the specific needs of LMICs.

**Table 1:** Individual characteristics of patients with cardiovascular disease

Characteristics	With established CVD (*N* = 9821)	
	Unweighted, *n*	Weighted, %
*Age*		
18–34 years	2090	31.2
35–44 years	1941	19.9
45–54 years	2240	19.8
55–69 years	3550	29.1
**Sex**		
Male	3565	43.9
Female	6256	56.1
*Country economic status*		
Upper-middle	2212	14.8
Lower-middle	5876	67.4
Low	1733	17.9
*Region*		
Africa	2079	21.5
Americas	425	2.8
Western Pacific	1615	15.0
European	2881	11.8
Eastern Mediterranean	979	12.2
South-East Asia	1842	36.7
*Education*		
No schooling	1285	22.1
Primary	3149	40.4
Secondary or higher	5162	37.5
*Area of residence*		
Urban	3311	32.4
Rural	3141	67.6

CVD = cardiovascular. Proportions were recalculated based on weights provided by individual surveys and the proportion of each country’s population aged 18–69

## Electronic Supplementary Material

Below is the link to the electronic supplementary material.

Supplementary Material 1

## Data Availability

Data from this study will be shared and made publicly available. After passing the application process, survey files, codebooks and de-identified microdata can be downloaded from the WHO website: https://extranet.who.int/ncdsmicrodata/index.php/catalog/steps/?page=1&ps=15&repo=STEPS.

## Abbreviations

WHO: World Health Organization
CVD: Cardiovascular disease
LMICs: Low-income and middle-income countries
BMI: Body mass index
SDG: Sustainable development goal
HICs: High-income countries
LICs: Low-income countries
STEPS: Stepwise Approach to Surveillance
L-MICs: Lower-middle-income countries
UMICs: Upper-middle-income countries
