# Supplementary material for: Achievement of Guideline-Recommended Targets for Secondary Prevention of Cardiovascular Disease in 38 Low-Income and Middle-Income Countries
Source: J Epidemiol Glob Health. 2024 Jun 3;14(3):1022–31. doi: 10.1007/s44197-024-00251-3 (PMC11444037; doi:10.1007/s44197-024-00251-3)
Supplement: Supplementary file 1 — Supplementary Material 1 [file 44197_2024_251_MOESM1_ESM.docx]

**Supplementary Materials**

[**Appendix 1: Supplemental methods 2**](#_Toc143954485)

[**Appendix 2: Survey and subpopulation characteristics 4**](#_Toc143954490)

[Supplementary Table S1: Survey characteristics](#_Toc143954500) 4

[Supplementary Table S2: Characteristics of study subpopulation with prior cardiovascular disease (CVD)](#_Toc143954501) 6

**Appendix 3: Prevalence of treatment, metabolic and lifestyle targets for secondary prevention of CVD, by region and country 7**

[Supplementary Table S3: Prevalence of treatment, metabolic and lifestyle targets for secondary prevention of CVD](#_Toc143954500) 7

[Supplementary Table S4: Prevalence of treatment, metabolic and lifestyle targets for secondary prevention of CVD by region](#_Toc143954501) 8

[Supplementary Table S5: Prevalence of treatment, metabolic and lifestyle targets for secondary prevention of CVD by country](#_Toc143954501) 10

[**Appendix 4: Prevalence of guideline-recommended targets for all treatment, metabolic and lifestyle components in patients with CVD by World Bank income group and region**](#_Toc143954505) **15**

[Supplementary Table S6: Prevalence of guideline-recommended targets for all treatment, metabolic and lifestyle components in patients with CVD by World Bank income group and region](#_Toc143954501) 15

**Appendix 5: Association between individual characteristics and guideline-recommended treatment, metabolic and lifestyle targets for patients with CVD 16**

Supplementary Table S7: Association between individual characteristics and guideline-recommended treatment, metabolic and lifestyle targets for patients with CVD (Univariate Poisson regression model) 16

Supplementary Table S8: Association between individual characteristics and guideline-recommended treatment, metabolic and lifestyle targets for patients with CVD (Multivariable Poisson regression model) 22

[**Appendix 6: Sensitivity Analysis**](#_Toc143954509) **28**

**[Appendix 7: STROBE Checklist](#_Toc143954509) 34**

**Appendix 1: Supplemental methods**

The following content complements the methodology section in the main paper.

**Inclusion criteria for surveys**

1. Since the STEP-wise approach to noncommunicable disease risk factor surveillance (STEPS) after 2013 introduced the question of cardiovascular disease (CVD) history, we selected the survey conducted after 2013. If there are multiple surveys for a country, the most recent one is selected. In addition, the latest research on secondary prevention of CVD in low- and middle-income countries (LMICs) comes from the Prospective Urban Rural Epidemiological (PURE) study, whose investigation date was before 2009. We wanted to update the status of secondary prevention in LMICs following the PURE study.
2. The surveys and studies are all obtained from the STEPS database on the World Health Organization (WHO) official website and have nationally representative individual-level data.
3. Surveys were exclusively carried out in countries classified as upper-middle (UMIC), lower-middle (L-MIC), or low-income (LIC) according to the World Bank's classification for the survey year.
4. For some countries, such as Pakistan and Tonga, they were excluded from our analysis because metabolic biochemical indicators were not collected.
5. Considering the safety of medication in pregnant women, as well as its prevalence in the real world, we excluded this group.

**Country classification and characteristics**

We grouped countries according to geographical regions defined by the WHO and income groups defined by the World Bank in the year the survey was conducted.

**Survey introduction**

For detailed sampling methods, survey procedures, WHO STEPS questionnaire and introductions for each country, please refer to the following websites (accessed January 3, 2023):

<https://extranet.who.int/ncdsmicrodata/index.php/catalog/steps/?page=1&ps=15&repo=STEPS>

**Data sharing**

Data from the 38 countries surveyed in this study are shared and publicly available. After passing the application process, survey files, codebooks and de-identified microdata can be downloaded via the link above or WHO website.

**Text of relevant questions in the underlying surveys**

1. History of cardiovascular diseases

“Have you ever had a heart attack or chest pain from heart disease (angina) or a stroke (cerebrovascular accident or incident)?”

1. Treatment targets

Aspirin: “Are you currently taking aspirin regularly to prevent or treat heart disease?”

Statins: “Are you currently taking statins (Lovastatin/Simvastatin/Atorvastatin or any other statin) regularly to prevent or treat heart disease?”

Antihypertensive drugs: “In the past two weeks, have you taken any drugs (medication) for raised blood pressure prescribed by a doctor or other health worker?”

1. Lifestyle targets

Tobacco use: “Do you currently smoke any tobacco products, such as cigarettes, cigars or pipes?”

Alcohol consumption: “Have you consumed any alcohol within the past 30 days?”

Dietary fruit and vegetable: “In a typical week, on how many days do you eat fruit?” & “How many servings of fruit do you eat on one of those days?” & “In a typical week, on how many days do you eat vegetables?” & “How many servings of vegetables do you eat on one of those days?”

Physical activity: “Does your work involve vigorous-intensity activity that causes large increases in breathing or heart rate like [carrying or lifting heavy loads, digging or construction work] for at least 10 minutes continuously?” & “In a typical week, on how many days do you do vigorous-intensity activities as part of your work?” & “How much time do you spend doing vigorous-intensity activities at work on a typical day?” & “Does your work involve moderate-intensity activity, that causes small increases in breathing or heart rate such as brisk walking [or carrying light loads] for at least 10 minutes continuously?” & “In a typical week, on how many days do you do moderate-intensity activities as part of your work?” & “How much time do you spend doing moderate-intensity activities at work on a typical day?”. Physical activities included work and recreational activities. The content of the Recreational activity questionnaire is the same as that of work.

**Statistical analysis**

Continuous variables are presented using means and standard deviations, while categorical variables are conveyed through counts and proportions. For each country in the STEPS dataset, the proportions of CVD secondary prevention behaviors, along with 95% confidence intervals, were computed using sampling weights, stratifications, and primary sampling units provided by the 38 countries. To refine the analysis, new adjusted weights were calculated for each country by utilizing the original sampling weights in the dataset (e.g., Step 1 weights from STEP Survey 1). These adjusted weights were derived by dividing the sum of the original weights by the 2019 population aged 18-69, and then multiplying the result by the original weights for each country.

In order to explore individualized characteristics influencing guideline compliance, we computed overall proportions and subgroups (e.g., gender, age groups: 18–34 years, 35–44 years, 45–54 years, 55–69 years. Proportion of secondary prevention recommendations by World Bank income category, WHO region, and education group). Due to the relatively low prevalence of our indicator of interest among CVD patients within each country, Zou's modified Poisson regression with robust error variance was employed to fit the multi-factor regression model. The treatment variables, metabolic variables, lifestyle variables, and covariates (such as gender and education) that were primarily analyzed had missing values of less than 0.5%. Consequently, we consider these missing data negligible, and they were not separately addressed in the statistical analyses.

We plotted Figures 1 to 3 using the ggplot2 package in R version 4.2.2. Other analyzes were performed in Stata version 18.0 software, mainly using the “svyset” command.

**Appendix 2: Survey and subpopulation characteristics**

Supplementary Table S1: Survey characteristics

|  | ISO code | Income group* | Year† | Response rate‡ | Sample size§ | Proportion of female participants (%) | Median age, years | With established CVD | Proportion of individuals by country with a history of CVD¶ | 2019 population ages 18-69 years (thousands) |
| --- | --- | --- | --- | --- | --- | --- | --- | --- | --- | --- |
| **Africa** |  |  |  |  |  |  |  |  |  |  |
| Algeria | DZA | UMIC | 2016 | 94 | 2584 | 55.3 | 40 (31-51) | 163 | 4.98 (3.68, 6.71) | 26,664 |
| Benin | BEN | LIC | 2015 | 99 | 4691 | 52.5 | 36 (27-46) | 270 | 1.09 (0.81, 1.47) | 5856 |
| Botswana | BWA | UMIC | 2014 | 64 | 2812 | 67.8 | 35 (26-48) | 157 | 0.27 (0.20, 0.36) | 1450 |
| Cabo Verde | CPV | L-MIC | 2020 | 64 | 2217 | 60.9 | 41 (30-53) | 88 | 0.07 (0.05, 0.09) | 354 |
| Eswatini | SWZ | L-MIC | 2014 | 76 | 2202 | 64.3 | 36 (26-50) | 90 | 0.12 (1.73, 2.32) | 633 |
| Ethiopia | ETH | LIC | 2015 | 96 | 7785 | 57.9 | 35 (25-45) | 276 | 9.69 (7.72, 12.10) | 51,881 |
| Kenya | KEN | LIC | 2015 | 92 | 3918 | 59.3 | 35 (27-47) | 266 | 4.96 (3.70, 6.62) | 26,574 |
| Malawi | MWI | LIC | 2017 | NR | 3549 | 64.4 | 36 (28-49) | 279 | 1.64 (1.25, 2.17) | 8805 |
| Sao Tome and Principe | STP | L-MIC | 2019 | 91 | 1726 | 59.7 | 36 (27-47) | 59 | 0.02 (0.01, 0.03) | 112 |
| Uganda | UGA | LIC | 2014 | 99 | 3319 | 58.0 | 33 (25-45) | 309 | 3.44 (2.78, 4.26) | 18,424 |
| Zambia | ZMB | L-MIC | 2017 | 74 | 3104 | 60.8 | 35 (25-46) | 122 | 1.67 (1.28, 2.19) | 8962 |
| **Americas** |  |  |  |  |  |  |  |  |  |  |
| Ecuador | ECU | UMIC | 2018 | 69 | 3899 | 58.0 | 40 (29-53) | 354 | 2.00 (1.73, 2.32) | 10,723 |
| Guyana | GUY | L-MIC | 2016 | 77 | 813 | 62.1 | 41 (29-53) | 71 | 0.09 (0.07, 0.12) | 492 |
| **Eastern Mediterranean** | |  |  |  |  |  |  |  |  |  |
| Afghanistan | AFG | LIC | 2018 | 78 | 3275 | 45.2 | 37 (25-49) | 244 | 3.33 (1.94, 5.68) | 17,845 |
| Iraq | IRQ | UMIC | 2015 | 99 | 3346 | 59.7 | 39 (29-50) | 171 | 4.62 (3.78, 5.64) | 24,736 |
| Jordan | JOR | UMIC | 2019 | 95 | 3157 | 63.9 | 40 (30-52) | 277 | 1.29 (1.07, 1.55) | 6916 |
| Lebanon | LBN | UMIC | 2017 | 66-74 | 1002 | 62.9 | 49 (39-56) | 52 | 0.61 (0.37, 0.99) | 3260 |
| Morocco | MAR | L-MIC | 2017 | 89 | 4139 | 65.7 | 43 (32-54) | 134 | 4.34 (3.60, 5.22) | 23,249 |
| Sudan | SDN | L-MIC | 2016 | 95 | 6358 | 63.7 | 37 (27-49) | 101 | 4.00 (2.92, 5.47) | 21,420 |
| **Europe** |  |  |  |  |  |  |  |  |  |  |
| Armenia | ARM | L-MIC | 2016 | 42 | 1540 | 71.6 | 46 (32-56) | 176 | 0.39 (0.30, 0.50) | 2086 |
| Azerbaijan | AZE | UMIC | 2017 | 97 | 2486 | 58.5 | 47 (34-57) | 204 | 1.35 (1.06, 1.72) | 7225 |
| Belarus | BLR | UMIC | 2016 | 87 | 4704 | 58.1 | 48 (36-58) | 367 | 1.24 (1.00, 1.54) | 6661 |
| Georgia | GEO | L-MIC | 2016 | 76 | 3044 | 72.1 | 51 (39-60) | 743 | 1.80 (1.54, 2.10) | 9622 |
| Kyrgyzstan | KGZ | LIC | 2013 | 100 | 2455 | 63.1 | 44 (34-53) | 355 | 0.74 (0.57, 0.95) | 3952 |
| Moldova | MDA | L-MIC | 2013 | 84 | 3427 | 64.1 | 49 (35-59) | 603 | 0.50 (0.42, 0.59) | 2671 |
| Tajikistan | TJK | L-MIC | 2016 | 94 | 2463 | 58.6 | 40 (29-50) | 133 | 1.04 (0.73, 1.49) | 5581 |
| Turkmenistan | TKM | UMIC | 2018 | 94 | 3730 | 57.0 | 40 (30-50) | 300 | 0.59 (0.47, 0.75) | 3174 |
| **Western Pacific** | |  |  |  |  |  |  |  |  |  |
| Kiribati | KIR | L-MIC | 2015 | 55 | 1078 | 55.5 | 37 (28-49) | 96 | 0.01 (0.01, 0.02) | 68 |
| Mongolia | MNG | L-MIC | 2019 | 98 | 5803 | 55.1 | 41 (32-53) | 948 | 0.40 (0.34, 0.47) | 2161 |
| Solomon Islands | SLB | L-MIC | 2015 | 58 | 1581 | 54.7 | 40 (31-50) | 137 | 0.06 (0.04, 0.09) | 345 |
| Tokelau | TK | UMIC | 2014 | 70 | 494 | 52.0 | 39 (27-51) | 44 | <0.01 | 0.8 |
| Tuvalu | TUV | UMIV | 2015 | 76 | 966 | 54.1 | 42 (29-55) | 123 | <0.01 | 7 |
| Vietnam | VNM | L-MIC | 2015 | 97 | 2959 | 56.6 | 44 (34-54) | 267 | 12.49 (10.49, 14.80) | 66867 |
| **South-East Asia** | |  |  |  |  |  |  |  |  |  |
| Bangladesh | BGD | L-MIC | 2018 | 84 | 6800 | 53.2 | 38 (30-48) | 745 | 18.48 (16.49, 20.65) | 98,963 |
| Myanmar | MMR | LIC | 2014 | 94 | 7540 | 65.4 | 45 (36-54) | 751 | 6.46 (4.46, 9.28) | 34,594 |
| Nepal | NPL | LIC | 2019 | 86 | 4860 | 64.4 | 40 (30-51) | 95 | 3.38 (2.35, 4.86) | 18,118 |
| Sir Lanka | LKA | L-MIC | 2014 | 72 | 4056 | 61.2 | 45 (34-55) | 222 | 2.68 (2.20, 3.26) | 14,339 |
| Timor-Leste | TLS | L-MIC | 2014 | 96 | 2224 | 57.8 | 40 (30-52) | 29 | 0.13 (0.08, 0.21) | 692 |
| **Total** | .. | .. | .. | 87 (74-95) | 126 106 | 59.9 | 40 (30-52) | 9821 |  |  |

World regions are defined by WHO. CVD=cardiovascular disease. ISO=International Organization for Standardization. UMIC=Upper-middle-income country. LIC=low-income country. L-MIC=lower middle-income country. *Income groups are defined by the World Bank fiscal year categories in the year the survey was done. †Year reflects the year(s) of survey data collection. ‡ Response rate is equal to the number of complete interviews divided by the total number of surveys. §The sample includes individuals aged 18-69 years of age. ¶ Proportion of individuals by country with a history of CVD was calculated by new weight based on each country’s 2019 population aged 18-69.

Supplementary Table S2: Characteristics of study subpopulation with prior CVD

| Characteristic | Low-income countries | Lower middle-income countries | Upper-middle-income countries | Overall |
| --- | --- | --- | --- | --- |
|  | n=9 countries | n=18 countries | n=11 countries | n=38 countries |
| Total No. | 1733 | 5876 | 2212 | 9821 |
| Age, median (IQR), years | 42 (30, 55) | 49 (37, 58) | 53 (41, 61) | 49 (36, 59) |
| Age group, years |  |  |  |  |
| 18-34 | 46.5 (42.2, 50.9) | 30.6 (27.7, 33.7) | 19.0 (15.5, 23.2) | 31.8 (29.6, 34.1) |
| 35-44 | 22.7 (19.4, 26.3) | 20.8 (18.8, 23.1) | 17.4 (14.5, 20.7) | 20.6 (19.1, 22.3) |
| 45-54 | 16.1 (12.9, 19.9) | 20.3 (18.3, 22.5) | 21.2 (18.3, 24.4) | 19.7 (18.1, 21.3) |
| 55-69 | 14.7 (11.7, 18.2) | 28.1 (25.6, 30.8) | 42.4 (38.1, 46.7) | 27.9 (26.0, 29.9) |
| Sex |  |  |  |  |
| Female | 51.9 (46.1, 57.7) | 59.2 (56.2, 62.2) | 47.0 (43.3, 50.8) | 55.7 (53.4, 58.0) |
| Male | 48.1 (42.3, 53.9) | 40.8 (37.8, 43.8) | 53.0 (49.2, 56.7) | 44.3 (42.0, 46.6) |
| Education |  |  |  |  |
| No formal schooling | 37.5 (35.6, 39.3) | 14.6 (13.8, 15.5) | 9.6 (8.7, 10.5) | 18.4 (17.6, 19.2) |
| Primary | 43.2 (41.4, 45.0) | 39.9 (38.6, 41.2) | 38.8 (37.4, 40.1) | 40.4 (39.5, 41.3) |
| Secondary or higher | 19.4 (18.1, 20.7) | 45.5 (44.0, 46.9) | 51.7 (50.0, 53.3) | 41.2 (40.2, 42.2) |
| Hypertension |  |  |  |  |
| Yes | 39.4 (34.9, 44.0) | 45.5 (42.5, 48.6) | 63.1 (58.5, 67.4) | 47.3 (45.0, 49.6) |
| Diabetes |  |  |  |  |
| Yes | 7.6 (5.4, 10.5) | 14.5 (12.4, 16.9) | 29.5 (25.9, 33.4) | 15.7 (14.1, 17.4) |
| Hypercholesterolemia |  |  |  |  |
| Yes | 7.3 (5.1, 10.3) | 16.7 (14.5, 19.1) | 36.7 (32.9, 40.7) | 18.2 (16.5, 20.0) |

Sample weights are re-adjusted based on each country’s 2019 population aged 18-69.

**Appendix 3:** **Prevalence of treatment, metabolic and lifestyle targets for secondary prevention of CVD, by region and country**

Supplementary Table S3: Prevalence of treatment, metabolic and lifestyle targets for secondary prevention of CVD

|  | Prevalence (95% CI) | | | |
| --- | --- | --- | --- | --- |
|  | Overall | Upper-Middle Income Countries | Lower-Middle Income Countries | Low-Income Countries |
| 1. **Treatment targets** | | | | |
| Antihypertensive drug | 22.70 (20.97-24.51) | 39.89 (35.68-44.24) | 21.62 (19.39-24.03) | 11.47 (8.74-14.92) |
| Statins | 13.63 (12.00-15.44) | 23.12 (19.76-26.86) | 12.31 (10.25-14.72) | 9.73 (6.46-14.40) |
| Aspirin | 19.60 (17.89-21.43) | 41.08 (36.85-45.45) | 15.83 (13.75-18.16) | 13.27 (9.89-17.59) |
| 1. **Metabolic targets** | | | | |
| Body mass index <25 kg/m² | 54.90 (52.52-57.25) | 26.62 (23.05-30.49) | 55.71 (52.70-58.67) | 76.40 (72.15-80.18) |
| Blood pressure <130/80 mm Hg | 39.92 (37.66-42.23) | 31.03 (27.10-35.25) | 42.69 (39.65-45.78) | 38.73 (33.92-43.77) |
| Total cholesterol <4.0 mmol/L | 46.09 (43.61-48.59) | 38.63 (34.75-42.67) | 41.18 (38.15-44.27) | 67.98 (63.11-72.50) |
| Fasting blood glucose <6.1 mmol/L | 84.89 (83.10-86.51) | 74.53 (70.65-78.06) | 85.43 (82.98-87.59) | 91.97 (88.92-94.23) |
| 1. **Lifestyle targets** | | | | |
| Not currently smoking | 83.20 (81.54-84.73) | 77.58 (73.95-80.83) | 82.23 (79.98-84.28) | 91.06 (88.02-93.38) |
| Not currently drinking | 83.14 (81.24-84.87) | 88.82 (86.73-90.62) | 84.59 (82.16-86.74) | 73.71 (67.99-78.73) |
| Sufficient physical activity | 65.45 (63.13-67.69) | 40.71 (37.00-44.53) | 65.87 (62.76-68.85) | 85.16 (82.32-87.61) |
| Dietary fruit and vegetables ≥5 servings/day | 16.18 (14.50-18.01) | 18.58 (15.65-21.91) | 19.46 (17.07-22.10) | 3.75 (2.64-5.30) |

Sample weights are re-adjusted based on each country’s 2019 population aged 18-69. Bangladesh, Myanmar, Nepal, Sir Lanka and Timor-Leste have a body mass index target of less than 23 kg/m^2^.

Supplementary Table S4: Prevalence of treatment, metabolic and lifestyle targets for secondary prevention of CVD by region

Treatment targets

| Region | Prevalence (95% CI) | | | |
| --- | --- | --- | --- | --- |
|  | Antihypertensive drug | Aspirin | Statins | All 3 targets met |
| Africa | 8.25 (6.71, 10.09) | 8.78 (7.14, 10.76) | 5.28 (3.96, 7.02) | 2.33 (1.56, 3.45) |
| Americas | 22.16 (17.72, 27.35) | 14.89 (11.17, 19.58) | 10.16 (7.15, 14.26) | 3.49 (1.95, 6.19) |
| Western Pacific | 18.97 (14.60, 24.29) | 13.94 (10.29, 18.62) | 12.47 (8.51, 17.91) | 2.95 (1.61, 5.34) |
| European | 45.11 (42.15, 48.11) | 32.52 (29.56, 35.62) | 12.65 (10.58, 15.07) | 7.33 (5.90, 9.08) |
| Eastern Mediterranean | 38.02 (32.75, 43.58) | 39.19 (33.89, 44.76) | 24.45 (19.64, 29.99) | 13.77 (10.36, 18.08) |
| South-East Asia | 22.79 (19.44, 26.54) | 17.38 (14.00, 21.37) | 15.76 (12.24, 20.06) | 6.31 (4.05, 9.72) |

Sample weights are re-adjusted based on each country’s 2019 population aged 18-69.

Metabolic targets

| Region | Prevalence (95% CI) | | | | |
| --- | --- | --- | --- | --- | --- |
|  | Body mass index <25 kg/m² | Blood pressure <130/80 mm Hg | Total cholesterol <4.0 mmol/L | Fasting blood-glucose <6.1 mmol/L | All 4 targets met |
| Africa | 72.37 (68.76, 75.70) | 45.11 (41.39, 48.90) | 67.48 (63.67, 71.07) | 91.80 (89.63, 93.54) | 27.45 (23.73, 31.51) |
| Americas | 31.75 (26.35, 37.69) | 56.65 (50.47, 62.62) | 40.39 (34.12, 46.99) | 83.67 (79.12, 87.39) | 12.44 (8.90, 17.13) |
| Western Pacific | 78.51 (72.62, 83.42) | 50.89 (43.47, 58.26) | 27.71 (21.66, 34.70) | 94.15 (88.83, 97.02) | 14.52 (9.97, 20.69) |
| European | 25.64 (22.68, 28.84) | 18.48 (16.17, 21.03) | 32.83 (29.82, 35.98) | 80.25 (77.74, 82.54) | 4.03 (3.00, 5.41) |
| Eastern Mediterranean | 40.03 (34.13, 46.24) | 26.97 (22.85, 31.53) | 50.02 (44.20, 55.83) | 74.55 (69.51, 79.00) | 11.91 (8.82, 15.89) |
| South-East Asia | 46.80 (42.49, 51.16) | 42.40 (37.83, 47.11) | 35.89 (31.78, 40.22) | 82.08 (77.87, 85.65) | 12.01 (9.40, 15.22) |

Sample weights are re-adjusted based on each country’s 2019 population aged 18-69. Bangladesh, Myanmar, Nepal, Sir Lanka and Timor-Leste have a body mass index target of less than 23 kg/m^2^.

Lifestyle targets

| Region | Prevalence (95% CI) | | | | |
| --- | --- | --- | --- | --- | --- |
|  | Not currently smoking | Not currently drinking | Sufficient physical activity | Fruit and vegetables ≥5 servings/day | All 4 targets met |
| Africa | 88.50 (85.79, 90.75) | 76.76 (72.43, 80.60) | 81.16 (77.77, 84.15) | 6.84 (5.30, 8.78) | 4.03 (2.92, 5.53) |
| Americas | 84.59 (79.90, 88.34) | 60.82 (54.68, 66.64) | 67.05 (61.31, 72.32) | 4.89 (2.89, 8.13) | 1.24 (0.38, 3.93) |
| Western Pacific | 77.47 (70.79, 82.99) | 62.86 (55.71, 69.49) | 57.18 (49.24, 64.77) | 43.28 (36.36, 50.48) | 12.49 (8.49, 18.01) |
| European | 80.63 (78.09, 82.95) | 72.32 (69.49, 74.99) | 50.67 (47.34, 54.00) | 30.30 (27.52, 33.23) | 11.59 (9.89, 13.53) |
| Eastern Mediterranean | 85.16 (81.07, 88.49) | 98.33 (96.54, 99.20) | 49.64 (44.02, 55.26) | 16.62 (12.70, 21.45) | 6.49 (3.91, 10.59) |
| South-East Asia | 80.22 (76.90, 83.16) | 92.58 (89.67, 94.72) | 67.54 (63.03, 71.75) | 10.31 (7.98, 13.22) | 6.44 (4.80, 8.60) |

Sample weights are re-adjusted based on each country’s 2019 population aged 18-69.

Supplementary Table S5: Prevalence of treatment, metabolic and lifestyle targets for secondary prevention of CVD by country

Treatment targets

| Country | Prevalence (95% CI) | | | |
| --- | --- | --- | --- | --- |
|  | Antihypertensive drug | Aspirin | Statins | All 3 targets met |
| Afghanistan | 37.33 (22.31, 55.28) | 44.21 (26.29, 63.78) | 40.77 (19.78, 65.77) | 24.85 (11.67, 45.30) |
| Algeria | 23.47 (17.42, 30.83) | 23.60 (17.34, 31.25) | 15.29 (10.38, 21.96) | 9.20 (5.47, 15.08) |
| Armenia | 32.84 (24.84, 41.97) | 29.52 (21.84, 38.56) | 7.23 (3.67, 13.78) | 3.74 (1.47, 9.16) |
| Azerbaijan | 45.73 (37.89, 53.78) | 41.09 (32.27, 50.52) | 21.55 (14.16, 31.38) | 9.91 (5.42, 17.44) |
| Bangladesh | 21.80 (17.05, 27.42) | 19.90 (14.76, 26.30) | 15.96 (10.82, 22.91) | 6.65 (3.35, 12.77) |
| Belarus | 61.52 (55.09, 67.58) | 50.32 (43.01, 57.61) | 17.90 (13.22, 23.78) | 14.44 (10.17, 20.11) |
| Benin | 5.98 (3.18, 10.95) | 10.73 (6.23, 17.87) | 5.36 (2.54, 10.96) | 1.20 (0.20, 6.71) |
| Botswana | 11.62 (6.88, 18.97) | 5.07 (2.20, 11.26) | 8.87 (2.03, 31.41) | 1.97 (0.37, 9.90) |
| Cabo Verde | 27.20 (17.12, 40.32) | 19.09 (11.21, 30.62) | 12.43 (6.31, 23.02) | 6.98 (2.95, 15.65) |
| Ecuador | 21.93 (17.34, 27.34) | 14.44 (10.61, 19.35) | 10.35 (7.21, 14.63) | 3.56 (1.95, 6.39) |
| Eswatini | 28.58 (15.77, 46.11) | 11.23 (4.63, 24.76) | 2.64 (0.59, 11.10) | <0.01 |
| Ethiopia | 3.32 (1.75, 6.21) | 4.32 (2.38, 7.69) | 3.45 (1.57, 7.40) | 0.85 (0.26, 2.73) |
| Georgia | 40.00 (35.41, 44.77) | 15.78 (12.63, 19.53) | 5.66 (3.93, 8.09) | 3.28 (2.03, 5.26) |
| Guyana | 27.13 (16.61, 41.05) | 24.76 (15.59, 36.97) | 6.17 (2.44, 14.73) | 2.03 (0.61, 6.48) |
| Iraq | 56.44 (46.51, 65.88) | 61.82 (52.60, 70.26) | 35.63 (27.06, 45.23) | 20.30 (13.26, 29.79) |
| Jordan | 42.94 (34.43, 51.89) | 53.68 (43.83, 63.26) | 30.04 (22.47, 38.88) | 24.04 (17.36, 32.29) |
| Kenya | 3.38 (1.50, 7.43) | 2.76 (1.16, 6.45) | 0.97 (0.32, 2.94) | 0.46 (0.08, 2.62) |
| Kiribati | 9.07 (4.32, 18.05) | 26.90 (16.10, 41.36) | 3.64 (1.51, 8.52) | 1.92 (0.57, 6.21) |
| Kyrgyzstan | 36.82 (30.77, 43.30) | 28.07 (22.60, 34.26) | 4.60 (2.55, 8.16) | 1.97 (0.65, 5.80) |
| Lebanon | 44.22 (24.11, 66.43) | 62.23 (39.22, 80.79) | 39.30 (21.07, 61.09) | 32.16 (16.75, 52.75) |
| Malawi | 7.62 (4.49, 12.63) | 11.22 (7.32, 16.82) | 5.78 (3.23, 10.13) | 2.29 (0.93, 5.53) |
| Moldova | 40.36 (35.36, 45.57) | 29.34 (25.10, 33.97) | 11.72 (8.41, 16.11) | 4.63 (3.04, 6.99) |
| Mongolia | 27.69 (24.35, 31.31) | 25.08 (22.13, 28.28) | 7.91 (5.94, 10.46) | 2.20 (1.45, 3.33) |
| Morocco | 25.27 (18.14, 34.05) | 12.69 (7.61, 20.43) | 9.58 (5.67, 15.74) | 2.75 (1.02, 7.18) |
| Myanmar | 22.45 (17.62, 28.14) | 9.17 (6.40, 12.98) | 7.82 (5.65, 10.71) | 2.24 (1.31, 3.82) |
| Nepal | 12.46 (5.47, 25.93) | 6.32 (2.43, 15.45) | 8.62 (2.94, 22.67) | <0.01 |
| Sao Tome and Principe | 24.84 (13.26, 41.68) | 23.60 (12.05, 41.05) | 10.70 (3.39, 29.02) | 7.50 (1.55, 29.52) |
| Solomon Islands | 4.15 (2.00, 8.41) | 8.86 (4.77, 15.87) | 2.56 (0.88, 7.21) | 2.03 (0.58, 6.87) |
| Sri Lanka | 42.97 (35.21, 51.09) | 34.06 (26.69, 42.30) | 42.77 (34.26, 51.74) | 21.96 (15.99, 29.37) |
| Sudan | 28.62 (17.96, 42.33) | 29.44 (19.59, 41.69) | 10.02 (5.07, 18.84) | 2.84 (0.93, 8.34) |
| Tajikistan | 41.97 (30.34, 54.57) | 16.75 (8.88, 29.35) | 8.66 (3.86, 18.30) | 3.05 (1.30, 7.00) |
| Timor-Leste | 35.25 (17.73, 57.91) | 10.36 (3.39, 27.59) | 10.78 (4.61, 23.17) | 2.26 (0.39, 11.90) |
| Tokelau | 17.71 (16.03, 19.53) | 13.75 (6.70, 26.13) | 5.73 (3.72, 8.72) | 3.84 (1.76, 8.19) |
| Turkmenistan | 52.67 (44.69, 60.52) | 64.36 (57.49, 70.69) | 23.95 (17.84, 31.37) | 17.68 (12.07, 25.16) |
| Tuvalu | 11.39 (6.42, 19.40) | 14.21 (10.62, 18.76) | 5.01 (2.93, 8.45) | 1.49 (0.50, 4.39) |
| Uganda | 7.54 (4.74, 11.79) | 7.14 (4.10, 12.13) | 1.72 (0.61, 4.72) | 0.39 (0.06, 2.65) |
| Vietnam | 18.78 (14.26, 24.32) | 13.59 (9.84, 18.48) | 12.68 (8.58, 18.34) | 2.98 (1.59, 5.48) |
| Zambia | 6.53 (3.35, 12.36) | 7.92 (3.76, 15.93) | 4.99 (2.21, 10.86) | 0.67 (0.16, 2.74) |

The estimates account for weighting and survey design.

Metabolic targets

| Country | Prevalence (95% CI) | | | | |
| --- | --- | --- | --- | --- | --- |
|  | Body mass index <25 kg/m² | Blood pressure <130/80 mm Hg | Total cholesterol <4.0 mmol/L | Fasting blood-glucose <6.1 mmol/L | All 4 targets met |
| Afghanistan | 56.04 (41.34, 69.75) | 20.35 (15.24,26.63) | 47.26 (28.03, 67.33) | 77.88 (61.17, 88.72) | 10.34 (5.82,17.72) |
| Algeria | 38.32 (30.76, 46.48) | 49.50 (40.79,58.24) | 49.25 (41.31, 57.24) | 78.74 (71.13, 84.78) | 15.56 (10.10,23.21) |
| Armenia | 32.87 (23.80, 43.43) | 23.03 (15.20,33.31) | 32.25 (23.32, 42.70) | 87.56 (80.47, 92.32) | 4.05 (1.18,13.01) |
| Azerbaijan | 24.76 (17.03, 34.53) | 26.18 (18.29,35.97) | 28.98 (20.93, 38.61) | 73.03 (65.66, 79.32) | 3.89 (1.32,10.92) |
| Bangladesh | 45.72 (40.28, 51.26) | 50.52 (44.34,56.69) | 32.63 (27.60, 38.10) | 84.20 (78.16, 88.80) | 13.91 (10.40,18.37) |
| Belarus | 13.60 (10.17, 17.95) | 6.37 (4.12,9.73) | 19.51 (15.26, 24.60) | 82.04 (76.41, 86.56) | 1.16 (0.32,4.13) |
| Benin | 70.20 (58.14, 79.98) | 35.04 (26.50,44.66) | 60.46 (51.26, 68.97) | 82.11 (71.53, 89.35) | 19.70 (13.70,27.49) |
| Botswana | 64.75 (53.29, 74.74) | 43.13 (32.42,54.52) | 63.51 (50.60, 74.72) | 93.37 (87.07, 96.72) | 19.83 (12.13,30.71) |
| Cabo Verde | 53.98 (39.39, 67.92) | 43.07 (29.23,58.08) | 51.98 (36.82, 66.78) | 82.39 (69.57, 90.55) | 19.13 (8.55,37.42) |
| Ecuador | 31.24 (25.65, 37.42) | 56.84 (50.41,63.05) | 41.74 (35.21, 48.58) | 84.22 (79.47, 88.04) | 12.90 (9.20,17.79) |
| Eswatini | 48.92 (32.58, 65.49) | 29.95 (18.33,44.88) | 55.54 (43.13, 67.30) | 83.83 (60.44, 94.62) | 11.40 (5.17,23.27) |
| Ethiopia | 89.19 (82.71, 93.44) | 44.96 (36.93,53.27) | 77.40 (70.70, 82.93) | 95.93 (90.97, 98.22) | 35.00 (27.16,43.74) |
| Georgia | 24.35 (20.24, 28.99) | 28.73 (24.45,33.42) | 36.82 (31.99, 41.93) | 88.80 (85.75, 91.26) | 5.42 (3.54,8.22) |
| Guyana | 42.98 (29.26, 57.87) | 52.42 (37.83,66.61) | 10.92 (4.99, 22.23) | 71.81 (58.10, 82.39) | 2.50 (0.59,9.97) |
| Iraq | 13.28 (8.77, 19.62) | 5.78 (2.65,12.13) | 26.68 (18.93, 36.18) | 60.94 (50.73, 70.28) | 0.00 (0.00,0.00) |
| Jordan | 23.25 (15.74, 32.94) | 40.94 (31.87,50.67) | 66.29 (57.52, 74.06) | 83.81 (76.88, 88.96) | 13.76 (7.78,23.17) |
| Kenya | 71.04 (65.08, 76.36) | 40.68 (33.52,48.26) | 61.57 (51.05, 71.10) | 97.03 (93.66, 98.64) | 21.27 (14.42,30.22) |
| Kiribati | 13.61 (5.82, 28.63) | 22.11 (12.37,36.33) | 42.45 (26.38, 60.29) | 60.86 (42.96, 76.26) | 2.73 (0.87,8.24) |
| Kyrgyzstan | 27.91 (22.77, 33.71) | 12.65 (8.91,17.65) | 32.77 (26.44, 39.81) | 83.37 (77.72, 87.81) | 5.25 (2.87,9.42) |
| Lebanon | 37.61 (19.38, 60.20) | 42.58 (23.09,64.68) | 23.93 (13.37, 39.08) | 71.97 (52.12, 85.83) | 0.00 (0.00,0.00) |
| Malawi | 71.12 (60.58, 79.79) | 52.87 (45.24,60.36) | 69.21 (61.70, 75.82) | 97.87 (95.38, 99.03) | 35.16 (27.37,43.83) |
| Moldova | 27.51 (22.56, 33.07) | 16.40 (12.55,21.13) | 23.49 (18.63, 29.16) | 84.03 (80.44, 87.06) | 3.12 (1.61,5.94) |
| Mongolia | 44.73 (40.45, 49.08) | 50.87 (46.55,55.17) | 33.30 (29.79, 37.01) | 71.54 (68.13, 74.72) | 11.27 (8.88,14.22) |
| Morocco | 49.31 (40.00, 58.67) | 41.50 (32.35,51.28) | 69.32 (60.48, 76.94) | 76.93 (68.84, 83.42) | 23.36 (15.48,33.64) |
| Myanmar | 45.64 (35.71, 55.94) | 31.59 (24.71,39.39) | 21.38 (16.75, 26.86) | 84.22 (77.26, 89.34) | 6.73 (4.27,10.43) |
| Nepal | 59.09 (44.52, 72.22) | 32.10 (17.46,51.37) | 65.79 (51.10, 77.97) | 68.34 (50.81, 81.86) | 16.77 (7.25,34.19) |
| Sao Tome and Principe | 48.40 (35.71, 61.29) | 30.09 (17.52,46.59) | 7.19 (2.11, 21.76) | 81.84 (65.59, 91.42) | 1.91 (0.32,10.56) |
| Solomon Islands | 35.87 (25.79, 47.37) | 52.85 (43.18,62.32) | 24.27 (16.48, 34.23) | 92.00 (84.97, 95.90) | 8.09 (4.03,15.56) |
| Sri Lanka | 41.17 (33.57, 49.21) | 25.39 (19.36,32.54) | 55.04 (46.58, 63.23) | 78.87 (71.39, 84.80) | 4.87 (2.42,9.58) |
| Sudan | 53.29 (39.43, 66.67) | 34.32 (23.06,47.67) | 57.03 (43.38, 69.69) | 82.30 (71.37, 89.67) | 15.75 (7.93,28.88) |
| Tajikistan | 37.63 (25.38, 51.70) | 13.98 (8.11,23.03) | 48.84 (36.04, 61.80) | 69.72 (59.12, 78.56) | 5.85 (2.81,11.78) |
| Timor-Leste | 54.80 (30.70, 76.84) | 43.98 (26.12,63.55) | 48.12 (25.66, 71.36) | 100.00 (0.00, 0.00) | 26.92 (11.22,51.79) |
| Tokelau | 1.92 (0.23, 14.29) | 55.97 (39.82,70.96) | 27.67 (21.74, 34.50) | 61.63 (56.30, 66.70) | 0.00 (0.00,0.00) |
| Turkmenistan | 26.58 (20.71, 33.41) | 9.20 (6.13,13.59) | 37.58 (30.33, 45.43) | 73.67 (66.73, 79.60) | 2.24 (0.94,5.25) |
| Tuvalu | 14.81 (10.29, 20.84) | 27.97 (22.04,34.78) | 61.86 (50.79, 71.82) | 95.70 (94.14, 96.86) | 7.42 (3.18,16.37) |
| Uganda | 74.97 (67.44, 81.24) | 39.00 (31.62,46.92) | 70.91 (63.35, 77.47) | 96.62 (93.70, 98.21) | 26.28 (20.54,32.96) |
| Vietnam | 79.90 (73.71, 84.93) | 50.91 (43.21,58.56) | 27.53 (21.28, 34.81) | 94.92 (89.00, 97.74) | 14.68 (9.96,21.10) |
| Zambia | 81.40 (72.12, 88.10) | 59.26 (49.34,68.47) | 80.46 (69.98, 87.91) | 82.46 (72.07, 89.55) | 40.35 (31.70,49.65) |

The estimates account for weighting and survey design. Bangladesh, Myanmar, Nepal, Sir Lanka and Timor-Leste have a body mass index target of less than 23 kg/m^2^.

Lifestyle targets

| Country | Prevalence (95% CI) | | | | |
| --- | --- | --- | --- | --- | --- |
|  | Not currently smoking | Not currently drinking | Sufficient physical activity | Fruit and vegetables ≥5 servings/day | All 4 targets met |
| Afghanistan | 90.47 (74.81, 96.81) | 98.91 (92.16, 99.86) | 72.96 (64.12, 80.30) | 0.43 (0.07, 2.78) | 0.39 (0.05, 2.96) |
| Algeria | 79.96 (71.88, 86.17) | 98.07 (94.75, 99.31) | 45.72 (37.68, 53.99) | 14.43 (9.02, 22.29) | 7.67 (4.33, 13.23) |
| Armenia | 66.26 (55.19, 75.79) | 52.05 (42.05, 61.88) | 59.88 (49.34, 69.59) | 26.06 (17.65, 36.69) | 8.74 (5.12, 14.52) |
| Azerbaijan | 78.03 (69.72, 84.57) | 81.15 (72.77, 87.40) | 43.54 (33.61, 54.02) | 31.39 (24.15, 39.66) | 9.46 (5.54, 15.69) |
| Bangladesh | 78.51 (73.41, 82.86) | 99.18 (97.52, 99.73) | 70.99 (64.67, 76.59) | 7.79 (5.39, 11.14) | 6.54 (4.27, 9.90) |
| Belarus | 79.55 (73.49, 84.52) | 60.44 (53.25, 67.20) | 42.49 (36.09, 49.16) | 19.50 (15.10, 24.80) | 5.92 (3.67, 9.43) |
| Benin | 94.40 (90.00, 96.93) | 58.94 (49.84, 67.47) | 67.45 (58.56, 75.24) | 7.37 (4.05, 13.06) | 4.75 (2.52, 8.76) |
| Botswana | 83.89 (73.03, 90.92) | 70.71 (56.39, 81.84) | 59.26 (46.81, 70.63) | 3.52 (0.83, 13.73) | 1.69 (0.29, 9.15) |
| Cabo Verde | 95.89 (90.66, 98.25) | 57.49 (42.35, 71.35) | 46.55 (31.84, 61.88) | 21.64 (13.37, 33.09) | 5.56 (2.18, 13.45) |
| Ecuador | 84.54 (79.65, 88.42) | 60.87 (54.47, 66.91) | 68.21 (62.25, 73.63) | 4.92 (2.86, 8.34) | 1.29 (0.40, 4.11) |
| Eswatini | 83.00 (62.25, 93.53) | 73.24 (53.86, 86.52) | 82.22 (68.12, 90.91) | 14.87 (6.68, 29.89) | 9.07 (4.19, 18.54) |
| Ethiopia | 93.35 (88.68, 96.17) | 65.80 (55.70, 74.65) | 90.63 (86.15, 93.77) | 1.50 (0.42, 5.28) | 1.01 (0.17, 5.85) |
| Georgia | 72.02 (66.88, 76.65) | 66.58 (61.76, 71.07) | 49.35 (43.64, 55.07) | 33.33 (28.67, 38.34) | 11.66 (9.00, 14.97) |
| Guyana | 85.69 (73.02, 92.98) | 59.81 (45.45, 72.66) | 41.70 (29.45, 55.07) | 4.19 (1.45, 11.52) | 0.00 (0.00, 0.00) |
| Iraq | 76.87 (67.46, 84.20) | 99.00 (94.29, 99.83) | 18.03 (12.32, 25.60) | 20.41 (13.88, 28.97) | 4.12 (1.80, 9.16) |
| Jordan | 56.43 (46.87, 65.54) | 98.20 (92.79, 99.57) | 54.98 (46.02, 63.63) | 15.18 (9.74, 22.88) | 3.00 (1.27, 6.92) |
| Kenya | 87.54 (78.33, 93.17) | 79.94 (67.75, 88.31) | 94.96 (90.81, 97.29) | 7.69 (4.54, 12.72) | 5.58 (3.11, 9.83) |
| Kiribati | 54.09 (42.85, 64.93) | 94.38 (84.63, 98.09) | 40.80 (24.30, 59.68) | 0.18 (0.02, 1.38) | 0.18 (0.02, 1.38) |
| Kyrgyzstan | 78.12 (72.79, 82.65) | 68.71 (61.51, 75.11) | 61.82 (54.05, 69.02) | 24.09 (18.25, 31.08) | 8.60 (5.48, 13.26) |
| Lebanon | 59.21 (36.82, 78.34) | 86.86 (59.85, 96.70) | 10.64 (4.96, 21.36) | 22.94 (10.78, 42.30) | 0.68 (0.09, 4.89) |
| Malawi | 92.09 (85.41, 95.86) | 86.73 (79.66, 91.60) | 91.59 (85.58, 95.23) | 9.29 (4.95, 16.77) | 8.59 (4.37, 16.20) |
| Moldova | 86.98 (82.90, 90.21) | 45.03 (39.58, 50.61) | 68.25 (62.55, 73.46) | 31.57 (26.46, 37.18) | 9.67 (6.60, 13.97) |
| Mongolia | 71.16 (67.51, 74.55) | 58.09 (53.78, 62.28) | 69.67 (65.52, 73.53) | 15.54 (12.39, 19.32) | 5.01 (3.47, 7.18) |
| Morocco | 91.30 (83.88, 95.48) | 97.62 (90.76, 99.42) | 56.81 (47.61, 65.57) | 24.87 (17.42, 34.19) | 9.56 (4.93, 17.72) |
| Myanmar | 81.56 (76.44, 85.77) | 83.32 (73.52, 89.99) | 57.02 (49.32, 64.41) | 13.79 (7.46, 24.09) | 6.33 (3.76, 10.49) |
| Nepal | 80.75 (70.14, 88.22) | 83.91 (71.87, 91.41) | 81.11 (63.43, 91.40) | 2.15 (0.43, 10.15) | 2.15 (0.43, 10.15) |
| Sao Tome and Principe | 91.59 (82.15, 96.26) | 28.96 (16.22, 46.20) | 69.31 (54.82, 80.78) | 9.16 (18.06, 52.73) | 5.32 (1.80, 14.72) |
| Solomon Islands | 70.56 (59.91, 79.36) | 89.48 (80.67, 94.54) | 75.95 (66.86, 83.17) | 13.21 (8.82, 19.32) | 8.50 (4.91, 14.31) |
| Sri Lanka | 88.74 (83.43, 92.50) | 80.33 (73.34, 85.84) | 51.67 (44.07, 59.19) | 29.42 (22.42, 37.54) | 11.77 (7.43, 18.14) |
| Sudan | 96.85 (92.13, 98.77) | 99.63 (97.45, 99.95) | 63.13 (50.56, 74.13) | 16.26 (7.05, 33.19) | 13.00 (5.11, 29.31) |
| Tajikistan | 95.26 (88.97, 98.05) | 96.38 (88.74, 98.90) | 44.86 (31.57, 58.92) | 22.72 (13.36, 35.93) | 10.97 (5.37, 21.08) |
| Timor-Leste | 67.34 (49.68, 81.15) | 93.04 (79.28, 97.90) | 74.13 (54.82, 87.13) | 13.62 (5.07, 31.77) | 0.00 (0.00, 0.00) |
| Tokelau | 36.79 (33.77, 39.91) | 50.37 (40.95, 59.76) | 70.99 (50.17, 85.61) | 3.65 (1.66, 7.83) | 0.00 (0.00, 0.00) |
| Turkmenistan | 96.43 (93.23, 98.15) | 93.06 (88.81, 95.78) | 63.61 (55.03, 71.41) | 64.05 (54.95, 72.24) | 36.38 (29.45, 43.92) |
| Tuvalu | 58.11 (49.29, 66.43) | 77.24 (62.37, 87.42) | 80.33 (71.29, 87.04) | 2.44 (0.74, 7.69) | 0.77 (0.09, 6.29) |
| Uganda | 86.39 (79.57, 91.18) | 71.13 (64.84, 76.71) | 89.12 (84.26, 92.61) | 5.15 (2.89, 9.03) | 1.64 (0.66, 4.05) |
| Vietnam | 77.74 (70.76, 83.44) | 62.84 (55.41, 69.71) | 56.69 (48.48, 64.56) | 44.38 (37.20, 51.81) | 12.77 (8.61, 18.52) |
| Zambia | 86.45 (75.92, 92.81) | 83.43 (73.82, 89.99) | 78.26 (68.94, 85.38) | 12.38 (6.80, 21.48) | 5.98 (2.24, 15.02) |

The estimates account for weighting and survey design.

**Appendix 4: Prevalence of guideline-recommended targets for all treatment, metabolic and lifestyle components in patients with CVD by World Bank income group and region**

Supplementary Table S6: Prevalence of guideline-recommended targets for all treatment, metabolic and lifestyle components in patients with CVD by World Bank income group and region

By World Bank income group

|  | Prevalence (95% CI) | | | |
| --- | --- | --- | --- | --- |
|  | Overall | Upper-Middle Income Countries | Lower-Middle Income Countries | Low-Income Countries |
| Treatment targets all met | 6.14 (5.06-7.42) | 14.13 (11.40-17.39) | 4.36 (3.11-6.08) | 4.96 (2.88-8.43) |
| Metabolic targets all met | 16.03 (14.30-17.92) | 7.93 (5.93-10.52) | 14.60 (12.57-16.91) | 27.45 (22.85-32.58) |
| Lifestyle targets all met | 6.85 (5.82-8.04) | 6.26 (4.77-8.19) | 8.49 (6.99-10.28) | 2.13 (1.27-3.53) |

Sample weights are re-adjusted based on each country’s 2019 population aged 18-69. Treatment targets include antihypertensive medication, aspirin and statin use. Metabolic targets include BMI <25 kg/m^2^, blood pressure <130/80 mm Hg, blood glucose <6.1 mmol/L (110 mg/dL), and total cholesterol <4.0 mmol/L (152 mg/dL). Bangladesh, Myanmar, Nepal, Sir Lanka and Timor-Leste have a body mass index target of less than 23 kg/m^2^. Lifestyle targets include non-smoking, non-drinking, sufficient physical activity and rational diet. It is not possible to show the prevalence of achieving all secondary prevention targets for treatment, metabolism, and lifestyle because the number of patients who achieve all secondary prevention targets simultaneously is zero.

By region

|  | Prevalence (95% CI) | | | | | |
| --- | --- | --- | --- | --- | --- | --- |
|  | Africa | Americas | Western Pacific | Europe | Eastern Mediterranean | South-East Asia |
| Treatment targets all met | 2.33 (1.56-3.45) | 3.49 (1.95-6.19) | 2.95 (1.61-5.34) | 7.33 (5.90-9.08) | 13.77 (10.36-18.08) | 6.31 (4.05-9.72) |
| Metabolic targets all met | 27.45 (23.73-31.51) | 12.44 (8.90-17.13) | 14.52 (9.97-20.69) | 4.03 (3.00-5.41) | 11.91 (8.82-15.89) | 12.01 (9.40-15.22) |
| Lifestyle targets all met | 4.03 (2.92-5.53) | 1.24 (0.38-3.93) | 12.49 (8.49-18.01) | 11.59 (9.89-13.53) | 6.49 (3.91-10.59) | 6.44 (4.80-8.60) |

Sample weights are re-adjusted based on each country’s 2019 population aged 18-69.

**Appendix 5: Association between individual characteristics and guideline-recommended treatment, metabolic and lifestyle targets for patients with CVD**

Supplementary Table S7: Association between individual characteristics and guideline-recommended treatment, metabolic and lifestyle targets for patients with CVD (Univariate Poisson regression model)

Treatment targets

|  | Prevalence (%) | Estimated average marginal effect (95% CI) | *P* values | Risk ratio (95% CI) | *P* values |
| --- | --- | --- | --- | --- | --- |
| **Antihypertensive drug** | | | | | |
| **Age, years** |  |  |  |  |  |
| 18-34 | 6.5 (4.7 to 8.9) | 0 (ref) |  | 1 (ref) |  |
| 35-44 | 12.0 (9.8 to 14.6) | 4.78 (1.43 to 8.13) | 0.005 | 1.16 (1.13 to 2.33) | 0.009 |
| 45-54 | 27.8 (24.4 to 31.6) | 19.25 (15.20 to 23.31) | <0.0001 | 3.51 (2.51 to 4.90) | <0.0001 |
| 55-69 | 45.5 (41.6 to 49.3) | 30.95 (26.43 to 35.48) | <0.0001 | 5.03 (3.61 to 7.01) | <0.0001 |
| **Sex** |  |  |  |  |  |
| Male | 22.8 (20.3 to 25.4) | 0 (ref) |  | 1 (ref) |  |
| Female | 22.6 (20.4 to 25.1) | 2.81 (-0.48 to 6.11) | 0.094 | 1.13 (0.98 to 1.31) | 0.095 |
| **Education** |  |  |  |  |  |
| No schooling | 24.4 (20.1 to 29.3) | 0 (ref) |  | 1 (ref) |  |
| Primary | 20.7 (18.3 to 23.3) | -7.79 (-13.86 to -1.71) | 0.012 | 0.73 (0.59 to 0.92) | 0.007 |
| Secondary or higher | 23.6 (21.1 to 26.4) | -8.33 (-17.61 to -1.86) | 0.012 | 0.71 (0.56 to 0.91) | 0.007 |
| **Aspirin** | | | | | |
| **Age, years** |  |  |  |  |  |
| 18-34 | 8.2 (6.1 to 10.8) | 0 (ref) |  | 1 (ref) |  |
| 35-44 | 13.4 (11.0 to 16.2) | 4.42 (0.73 to 8.11) | 0.019 | 1.46 (1.05 to 2.02) | 0.024 |
| 45-54 | 21.4 (18.3 to 24.9) | 11.60 (7.88 to 15.31) | <0.0001 | 2.20 (1.65 to 2.93) | <0.0001 |
| 55-70 | 36.0 (32.2 to 40.0) | 20.13 (15.34 to 24.92) | <0.0001 | 3.08 (2.26 to 4.20) | <0.0001 |
| **Sex** |  |  |  |  |  |
| Male | 22.2 (19.7 to 24.8) | 0 (ref) |  | 1 (ref) |  |
| Female | 17.5 (15.3 to 20.1) | -1.00 (-4.19 to 2.19) | 0.539 | 0.95 (0.81 to 1.12) | 0.540 |
| **Education** |  |  |  |  |  |
| No schooling | 22.3 (17.9 to 27.5) | 0 (ref) |  | 1 (ref) |  |
| Primary | 17.9 (15.6 to 20.5) | -5.26 (-10.61 to 0.09) | 0.054 | 0.78 (0.61 to 0.99) | 0.041 |
| Secondary or higher | 19.6 (17.3 to 22.1) | -5.21 (-10.90 to 0.48) | 0.073 | 0.78 (0.60 to 1.01) | 0.059 |
| **Statins** | | | | | |
| **Age, years** |  |  |  |  |  |
| 18-34 | 6.5 (4.5 to 9.3) | 0 (ref) |  | 1 (ref) |  |
| 35-44 | 8.1 (6.0 to 10.8) | 0.93 (-2.45 to 4.31) | 0.589 | 1.12 (0.73 to 1.73) | 0.593 |
| 45-54 | 14.7 (12.0 to 18.0) | 7.20 (3.41 to 10.99) | <0.0001 | 1.96 (1.33 to 2.90) | 0.001 |
| 55-70 | 25.1 (21.5 to 29.1) | 13.94 (9.48 to 18.40) | <0.0001 | 2.87 (1.95 to 4.22) | <0.0001 |
| **Sex** |  |  |  |  |  |
| Male | 15.7 (13.3 to 18.4) | 0 (ref) |  | 1 (ref) |  |
| Female | 12.0 (9.9 to 14.4) | -1.14 (-4.31 to 2.02) | 0.478 | 0.92 (0.73 to 1.16) | 0.480 |
| **Education** |  |  |  |  |  |
| No schooling | 16.1 (11.7 to 21.7) | 0 (ref) |  | 1 (ref) |  |
| Primary | 13.2 (11.0 to 15.7) | -2.27 (-7.56 to 3.02) | 0.400 | 0.85 (0.59 to 1.22) | 0.379 |
| Secondary or higher | 12.9 (11.0 to 15.1) | -1.79 (-7.25 to 3.68) | 0.521 | 0.88 (0.61 to 1.28) | 0.508 |

Metabolic targets

|  | Prevalence (%) | Estimated average marginal effect (95% CI) | *P* values | Risk ratio (95% CI) | *P* values |
| --- | --- | --- | --- | --- | --- |
| **Body mass index <25 kg/m²** | | | | | |
| **Age, years** |  |  |  |  |  |
| 18-34 | 70.5 (66.4 to 74.4) | 0 (ref) |  | 1 (ref) |  |
| 35-44 | 54.6 (50.1 to 59.0) | -10.40 (-15.30 to -5.49) | <0.0001 | 0.84 (0.77 to 0.91) | <0.0001 |
| 45-54 | 47.1 (42.6 to 51.7) | -15.54 (-20.97 to -10.11) | <0.0001 | 0.76 (0.68 to 0.84) | <0.0001 |
| 55-69 | 42.7 (39.0 to 46.6) | -15.20 (-20.60 to -9.81) | <0.0001 | 0.76 (0.69 to 0.84) | <0.0001 |
| **Sex** |  |  |  |  |  |
| Male | 58.4 (55.1 to 61.6) | 0 (ref) |  | 1 (ref) |  |
| Female | 52.1 (49.1 to 55.1) | -8.92 (-12.71 to -5.13) | <0.0001 | 0.85 (0.79 to 0.91) | <0.0001 |
| **Education** |  |  |  |  |  |
| No schooling | 59.5 (54.6 to 64.3) | 0 (ref) |  | 1 (ref) |  |
| Primary | 54.4 (50.7 to 58.0) | -2.48 (-8.51 to 3.55) | 0.420 | 0.96 (0.86 to 1.06) | 0.417 |
| Secondary or higher | 52.9 (49.5 to 56.2) | -4.19 (-10.75 to 2.37) | 0.210 | 0.93 (0.82 to 1.04) | 0.207 |
| **Blood pressure <130/80 mm Hg** | | | | | |
| **Age, years** |  |  |  |  |  |
| 18-34 | 56.4 (52.2 to 60.5) | 0 (ref) |  | 1 (ref) |  |
| 35-44 | 42.8 (38.4 to 47.4) | -11.17 (-16.65 to -5.68) | <0.0001 | 0.79 (0.70 to 0.89) | <0.0001 |
| 45-54 | 32.2 (28.1 to 36.5) | -20.89 (-26.47 to -15.31) | <0.0001 | 0.61 (0.53 to 0.70) | <0.0001 |
| 55-70 | 24.5 (21.3 to 27.9) | -27.35 (-32.69 to -22.01) | <0.0001 | 0.49 (0.42 to 0.57) | <0.0001 |
| **Sex** |  |  |  |  |  |
| Male | 39.6 (36.4 to 42.8) | 0 (ref) |  | 1 (ref) |  |
| Female | 40.2 (37.2 to 43.3) | -1.69 (-5.72 to 2.35) | 0.413 | 0.96 (0.87 to 1.06) | 0.412 |
| **Education** |  |  |  |  |  |
| No schooling | 35.7 (31.3 to 40.2) | 0 (ref) |  | 1 (ref) |  |
| Primary | 40.3 (36.7 to 44.0) | 6.02 (0.50 to 11.53) | 0.032 | 1.18 (1.01 to 1.39) | 0.036 |
| Secondary or higher | 42.1 (38.8 to 45.4) | 13.70 (7.71 to 19.69) | <0.0001 | 1.42 (1.21 to 1.66) | <0.0001 |
| **Total cholesterol <4.0 mmol/L** | | | | | |
| **Age, years** |  |  |  |  |  |
| 18-34 | 62.4 (57.8 to 66.9) | 0 (ref) |  | 1 (ref) |  |
| 35-44 | 46.3 (41.7 to 51.0) | -11.91 (-17.21 to -6.61) | <0.0001 | 0.79 (0.71 to 0.88) | <0.0001 |
| 45-54 | 35.6 (31.4 to 40.1) | -19.14 (-24.86 to -13.43) | <0.0001 | 0.66 (0.58 to 0.76) | <0.0001 |
| 55-70 | 34.6 (31.2 to 38.2) | -18.34 (-23.65 to -13.03) | <0.0001 | 0.68 (0.60 to 0.76) | <0.0001 |
| **Sex** |  |  |  |  |  |
| Male | 52.0 (48.5 to 55.6) | 0 (ref) |  | 1 (ref) |  |
| Female | 41.4 (38.4 to 44.4) | -10.69 (-14.58 to -6.80) | <0.0001 | 0.79 (0.73 to 0.86) | <0.0001 |
| **Education** |  |  |  |  |  |
| No schooling | 49.7 (44.7 to 54.8) | 0 (ref) |  | 1 (ref) |  |
| Primary | 46.5 (42.7 to 50.4) | 2.82 (-2.14 to 7.78) | 0.265 | 1.07 (0.95 to 1.19) | 0.268 |
| Secondary or higher | 43.0 (39.4 to 46.6) | 4.80 (-1.21 to 10.81) | 0.117 | 1.11 (0.97 to 1.27) | 0.118 |
| **Fasting blood-glucose<6.1 mmol/L** | | | | | |
| **Age, years** |  |  |  |  |  |
| 18-34 | 92.5 (89.6 to 94.6) | 0 (ref) |  | 1 (ref) |  |
| 35-44 | 87.9 (85.0 to 90.3) | -2.69 (-6.16 to 0.78) | 0.129 | 0.97 (0.93 to 1.01) | 0.129 |
| 45-54 | 80.5 (76.8 to 83.7) | -10.02 (-14.13 to -5.91) | <0.0001 | 0.89 (0.85 to 0.93) | <0.0001 |
| 55-70 | 77.0 (73.2 to 80.4) | -12.21 (-16.85 to -7.58) | <0.0001 | 0.87 (0.82 to 0.92) | <0.0001 |
| **Sex** |  |  |  |  |  |
| Male | 84.4 (81.9 to 86.6) | 0 (ref) |  | 1 (ref) |  |
| Female | 85.3 (82.8 to 87.5) | -0.52 (-3.62 to 2.59) | 0.745 | 0.99 (0.96 to 1.03) | 0.745 |
| **Education** |  |  |  |  |  |
| No schooling | 83.0 (78.1 to 86.9) | 0 (ref) |  | 1 (ref) |  |
| Primary | 83.9 (81.3 to 86.3) | 1.81 (-2.99 to 6.61) | 0.461 | 1.02 (0.96 to 1.08) | 0.464 |
| Secondary or higher | 86.7 (84.4 to 88.7) | 4.75 (-0.57 to 10.06) | 0.080 | 1.06 (0.99 to 1.13) | 0.084 |

Bangladesh, Myanmar, Nepal, Sir Lanka and Timor-Leste have a body mass index target of less than 23 kg/m^2^.

Lifestyle targets

|  | Prevalence (%) | Estimated average marginal effect (95% CI) | *P* values | Risk ratio (95% CI) | *P* values |
| --- | --- | --- | --- | --- | --- |
| **Not currently smoking** | | | | | |
| **Age, years** |  |  |  |  |  |
| 18-34 | 85.5 (72.1 to 88.4) | 0 (ref) |  | 1 (ref) |  |
| 35-44 | 81.4 (77.6 to 84.6) | -3.43 (-8.07 to 1.21) | 0.147 | 0.98 (0.96 to 1.01) | 0.147 |
| 45-54 | 83.6 (80.6 to 86.3) | 0.10 (-4.37 to 4.57) | 0.965 | 1.00 (0.98 to 1.03) | 0.965 |
| 55-69 | 81.6 (78.5 to 82.3) | -0.92 (-5.55 to 3.71) | 0.697 | 1.00 (0.97 to 1.02) | 0.697 |
| **Sex** |  |  |  |  |  |
| Male | 66.1 (62.9 to 69.2) | 0 (ref) |  | 1 (ref) |  |
| Female | 96.8 (95.8 to 97.5) | 31.56 (28.52 to 34.60) | <0.0001 | 1.19 (1.17 to 1.21) | <0.0001 |
| **Education** |  |  |  |  |  |
| No schooling | 86.0 (82.7 to 88.8) | 0 (ref) |  | 1 (ref) |  |
| Primary | 80.4 (77.6 to 82.9) | -2.18 (-6.53 to 2.17) | 0.327 | 0.99 (0.96 to 1.01) | 0.326 |
| Secondary or higher | 83.8 (81.2 to 86.2) | 2.55 (-2.54 to 7.65) | 0.326 | 1.01 (0.99 to 1.04) | 0.327 |
| **Not currently drinking** | | | | | |
| **Age, years** |  |  |  |  |  |
| 18-34 | 82.8 (79.1 to 85.9) | 0 (ref) |  | 1 (ref) |  |
| 35-44 | 80.4 (76.1 to 84.0) | -4.02 (-8.54 to 0.51) | 0.082 | 0.98 (0.95 to 1.00) | 0.082 |
| 45-54 | 83.2 (79.8 to 86.1) | 0.22 (-4.01 to 4.44) | 0.920 | 1.00 (0.98 to 1.02) | 0.920 |
| 55-70 | 85.6 (83.0 to 87.8) | 0.54 (-3.25 to 4.33) | 0.780 | 1.00 (0.98 to 1.02) | 0.780 |
| **Sex** |  |  |  |  |  |
| Male | 72.7 (69.2 to 75.9) | 0 (ref) |  | 1 (ref) |  |
| Female | 91.5 (89.9 to 92.8) | 20.31 (17.23 to 23.38) | <0.0001 | 1.12 (1.10 to 1.14) | <0.0001 |
| **Education** |  |  |  |  |  |
| No schooling | 87.3 (83.4 to 90.4) | 0 (ref) |  | 1 (ref) |  |
| Primary | 85.9 (83.3 to 88.2) | 1.25 (-2.01 to 4.51) | 0.452 | 1.01 (0.99 to 1.02) | 0.453 |
| Secondary or higher | 77.0 (73.9 to 79.9) | -2.78 (-6.47 to 0.90) | 0.138 | 0.98 (0.97 to 1.00) | 0.138 |
| **Sufficient physical activity** | | | | | |
| **Age, years** |  |  |  |  |  |
| 18-34 | 76.8 (72.6 to 80.6) | 0 (ref) |  | 1 (ref) |  |
| 35-44 | 76.0 (72.1 to 79.4) | 3.51 (-1.15 to 8.18) | 0.140 | 1.05 (0.98 to 1.12) | 0.142 |
| 45-54 | 66.8 (62.8 to 70.6) | -2.00 (-7.25 to 3.26) | 0.456 | 0.97 (0.90 to 1.05) | 0.457 |
| 55-70 | 43.7 (40.0 to 47.5) | -22.37 (-27.86 to -16.88) | <0.0001 | 0.68 (0.62 to 0.75) | <0.0001 |
| **Sex** |  |  |  |  |  |
| Male | 65.0 (61.8 to 68.1) | 0 (ref) |  | 1 (ref) |  |
| Female | 65.8 (62.7 to 68.8) | -2.04 (-5.97 to 1.89) | 0.309 | 0.97 (0.91 to 1.03) | 0.309 |
| **Education** |  |  |  |  |  |
| No schooling | 69.6 (64.9 to 74.0) | 0 (ref) |  | 1 (ref) |  |
| Primary | 65.3 (61.7 to 68.8) | 1.52 (-3.69 to 6.72) | 0.568 | 1.02 (0.94 to 1.11) | 0.569 |
| Secondary or higher | 62.6 (59.1 to 65.9) | 2.28 (-4.01 to 8.57) | 0.478 | 1.04 (0.94 to 1.14) | 0.479 |
| **Dietary fruit and vegetables ≥5 servings/day** | | | | | |
| **Age, years** |  |  |  |  |  |
| 18-34 | 13.9 (10.9 to 17.6) | 0 (ref) |  | 1 (ref) |  |
| 35-44 | 14.4 (11.5 to 17.7) | -1.14 (-5.61 to 3.33) | 0.617 | 0.93 (0.71 to 1.22) | 0.614 |
| 45-54 | 15.8 (13.1 to 18.9) | -2.68 (-7.30 to 1.94) | 0.255 | 0.84 (0.63 to 1.12) | 0.245 |
| 55-70 | 20.4 (17.6 to 23.5) | -0.45 (-4.84 to 3.94) | 0.841 | 0.97 (0.75 to 1.26) | 0.840 |
| **Sex** |  |  |  |  |  |
| Male | 17.2 (14.7 to 20.1) | 0 (ref) |  | 1 (ref) |  |
| Female | 15.4 (13.4 to 17.6) | -1.79 (-4.76 to 1.18) | 0.237 | 0.90 (0.75 to 1.07) | 0.231 |
| **Education** |  |  |  |  |  |
| No schooling | 5.2 (3.9 to 7.1) | 0 (ref) |  | 1 (ref) |  |
| Primary | 13.3 (11.1 to 16.0) | 5.30 (1.81 to 8.79) | 0.003 | 1.62 (1.15 to 2.28) | 0.006 |
| Secondary or higher | 25.8 (22.7 to 29.1) | 11.74 (7.76 to 15.73) | <0.0001 | 2.37 (1.68 to 3.34) | <0.0001 |

Supplementary Table S7: Association between individual characteristics and guideline-recommended treatment, metabolic and lifestyle targets for patients with CVD (Multivariable Poisson regression model)

Treatment targets

|  | Prevalence (%) | Estimated average marginal effect (95% CI) | *P* values | Risk ratio (95% CI) | *P* values |
| --- | --- | --- | --- | --- | --- |
| **Antihypertensive drug** | | | | | |
| **Age, years** |  |  |  |  |  |
| 18-34 | 6.5 (4.7 to 8.9) | 0 (ref) |  | 1 (ref) |  |
| 35-44 | 12.0 (9.8 to 14.6) | 4.32 (1.06, 7.59) | 0.01 | 1.56 (1.09, 2.25) | 0.02 |
| 45-54 | 27.8 (24.4 to 31.6) | 19.00 (14.88, 23.12) | <0.0001 | 3.47 (2.47, 4.87) | <0.0001 |
| 55-69 | 45.5 (41.6 to 49.3) | 31.75 (26.99, 36.50) | <0.0001 | 5.13 (3.65, 7.21) | <0.0001 |
| **Sex** |  |  |  |  |  |
| Male | 22.8 (20.3 to 25.4) | 0 (ref) |  | 1 (ref) |  |
| Female | 22.6 (20.4 to 25.1) | 3.95 (0.89, 7.02) | 0.01 | 1.19 (1.04, 1.37) | 0.01 |
| **Education** |  |  |  |  |  |
| No schooling | 24.4 (20.1 to 29.3) | 0 (ref) |  | 1 (ref) |  |
| Primary | 20.7 (18.3 to 23.3) | 0.62 (-3.82, 5.07) | 0.78 | 1.03 (0.84, 1.27) | 0.79 |
| Secondary or higher | 23.6 (21.1 to 26.4) | 3.01 (-1.87, 7.89) | 0.23 | 1.14 (0.92, 1.42) | 0.23 |
| **Aspirin** | | | | | |
| **Age, years** |  |  |  |  |  |
| 18-34 | 8.2 (6.1 to 10.8) | 0 (ref) |  | 1 (ref) |  |
| 35-44 | 13.4 (11.0 to 16.2) | 4.57 (0.79, 8.35) | 0.02 | 1.47 (1.05, 2.04) | 0.02 |
| 45-54 | 21.4 (18.3 to 24.9) | 11.39 (7.59, 15.18) | <0.0001 | 2.16 (1.61, 2.89) | <0.0001 |
| 55-70 | 36.0 (32.2 to 40.0) | 19.59 (14.59, 24.59) | <0.0001 | 3.00 (2.18, 4.12) | <0.0001 |
| **Sex** |  |  |  |  |  |
| Male | 22.2 (19.7 to 24.8) | 0 (ref) |  | 1 (ref) |  |
| Female | 17.5 (15.3 to 20.1) | -0.50 (-3.54, 2.54) | 0.75 | 0.97 (0.83, 1.14) | 0.75 |
| **Education** |  |  |  |  |  |
| No schooling | 22.3 (17.9 to 27.5) | 0 (ref) |  | 1 (ref) |  |
| Primary | 17.9 (15.6 to 20.5) | -0.81 (-5.17, 3.55) | 0.72 | 0.96 (0.77, 1.20) | 0.71 |
| Secondary or higher | 19.6 (17.3 to 22.1) | 0.59 (-4.08, 5.25) | 0.81 | 1.03 (0.81, 1.30) | 0.81 |
| **Statins** | | | | | |
| **Age, years** |  |  |  |  |  |
| 18-34 | 6.5 (4.5 to 9.3) | 0 (ref) |  | 1 (ref) |  |
| 35-44 | 8.1 (6.0 to 10.8) | 1.17 (-2.31, 4.65) | 0.51 | 1.16 (0.74, 1.81) | 0.52 |
| 45-54 | 14.7 (12.0 to 18.0) | 7.52 (3.61, 11.44) | <0.0001 | 2.02 (1.34, 3.03) | <0.0001 |
| 55-70 | 25.1 (21.5 to 29.1) | 14.35 (9.63, 19.08) | <0.0001 | 2.94 (1.94, 4.45) | <0.0001 |
| **Sex** |  |  |  |  |  |
| Male | 15.7 (13.3 to 18.4) | 0 (ref) |  | 1 (ref) |  |
| Female | 12.0 (9.9 to 14.4) | -0.27 (-3.46, 2.93) | 0.87 | 0.98 (0.78, 1.24) | 0.87 |
| **Education** |  |  |  |  |  |
| No schooling | 16.1 (11.7 to 21.7) | 0 (ref) |  | 1 (ref) |  |
| Primary | 13.2 (11.0 to 15.7) | 0.59 (-3.59, 4.76) | 0.78 | 1.05 (0.76, 1.44) | 0.79 |
| Secondary or higher | 12.9 (11.0 to 15.1) | 2.07 (-2.85, 7.00) | 0.41 | 1.16 (0.81, 1.67) | 0.42 |

Metabolic targets

|  | Prevalence (%) | Estimated average marginal effect (95% CI) | *P* values | Risk ratio (95% CI) | *P* values |
| --- | --- | --- | --- | --- | --- |
| **Body mass index <25 kg/m²** | | | | | |
| **Age, years** |  |  |  |  |  |
| 18-34 | 70.5 (66.4 to 74.4) | 0 (ref) |  | 1 (ref) |  |
| 35-44 | 54.6 (50.1 to 59.0) | -11.68 (-16.80, -6.56) | <0.0001 | 0.82 (0.75, 0.90) | <0.0001 |
| 45-54 | 47.1 (42.6 to 51.7) | -17.00 (-22.79, -11.21) | <0.0001 | 0.74 (0.66, 0.82) | <0.0001 |
| 55-69 | 42.7 (39.0 to 46.6) | -17.66 (-23.32, -12.00) | <0.0001 | 0.73 (0.66, 0.81) | <0.0001 |
| **Sex** |  |  |  |  |  |
| Male | 58.4 (55.1 to 61.6) | 0 (ref) |  | 1 (ref) |  |
| Female | 52.1 (49.1 to 55.1) | -10.08 (-14.08, -6.07) | <0.0001 | 0.83 (0.78, 0.90) | <0.0001 |
| **Education** |  |  |  |  |  |
| No schooling | 59.5 (54.6 to 64.3) | 0 (ref) |  | 1 (ref) |  |
| Primary | 54.4 (50.7 to 58.0) | -8.73 (-15.12, -2.35) | 0.01 | 0.86 (0.78, 0.96) | 0.01 |
| Secondary or higher | 52.9 (49.5 to 56.2) | -13.55 (-20.75, -6.34) | <0.0001 | 0.79 (0.70, 0.89) | <0.0001 |
| **Blood pressure <130/80 mm Hg** | | | | | |
| **Age, years** |  |  |  |  |  |
| 18-34 | 56.4 (52.2 to 60.5) | 0 (ref) |  | 1 (ref) |  |
| 35-44 | 42.8 (38.4 to 47.4) | -10.24 (-15.96, -4.52) | <0.0001 | 0.81 (0.71, 0.91) | <0.0001 |
| 45-54 | 32.2 (28.1 to 36.5) | -19.26 (-25.27, -13.25) | <0.0001 | 0.63 (0.54, 0.74) | <0.0001 |
| 55-70 | 24.5 (21.3 to 27.9) | -26.33 (-32.04, -20.61) | <0.0001 | 0.50 (0.42, 0.59) | <0.0001 |
| **Sex** |  |  |  |  |  |
| Male | 39.6 (36.4 to 42.8) | 0 (ref) |  | 1 (ref) |  |
| Female | 40.2 (37.2 to 43.3) | -1.78 (-5.69, 2.14) | 0.37 | 0.96 (0.87, 1.05) | 0.370 |
| **Education** |  |  |  |  |  |
| No schooling | 35.7 (31.3 to 40.2) | 0 (ref) |  | 1 (ref) |  |
| Primary | 40.3 (36.7 to 44.0) | -0.12 (-5.99, 5.75) | 0.97 | 1.00 (0.86, 1.16) | 0.97 |
| Secondary or higher | 42.1 (38.8 to 45.4) | 4.28 (-2.44, 11.00) | 0.21 | 1.11 (0.94, 1.32) | 0.22 |
| **Total cholesterol <4.0 mmol/L** | | | | | |
| **Age, years** |  |  |  |  |  |
| 18-34 | 62.4 (57.8 to 66.9) | 0 (ref) |  | 1 (ref) |  |
| 35-44 | 46.3 (41.7 to 51.0) | -13.03 (-18.54, -7.52) | <0.0001 | 0.77 (0.69, 0.86) | <0.0001 |
| 45-54 | 35.6 (31.4 to 40.1) | -19.85 (-25.67, -14.03) | <0.0001 | 0.65 (0.57, 0.75) | <0.0001 |
| 55-70 | 34.6 (31.2 to 38.2) | -19.91 (-25.41, -14.41) | <0.0001 | 0.65 (0.58, 0.74) | <0.0001 |
| **Sex** |  |  |  |  |  |
| Male | 52.0 (48.5 to 55.6) | 0 (ref) |  | 1 (ref) |  |
| Female | 41.4 (38.4 to 44.4) | -11.81 (-15.73, -7.89) | <0.0001 | 0.77 (0.71, 0.84) | <0.0001 |
| **Education** |  |  |  |  |  |
| No schooling | 49.7 (44.7 to 54.8) | 0 (ref) |  | 1 (ref) |  |
| Primary | 46.5 (42.7 to 50.4) | -4.04 (-9.54, 1.47) | 0.15 | 0.92 (0.82, 1.03) | 0.15 |
| Secondary or higher | 43.0 (39.4 to 46.6) | -5.95 (-12.45, 0.54) | 0.07 | 0.88 (0.77, 1.01) | 0.07 |
| **Fasting blood-glucose <6.1 mmol/L** | | | | | |
| **Age, years** |  |  |  |  |  |
| 18-34 | 92.5 (89.6 to 94.6) | 0 (ref) |  | 1 (ref) |  |
| 35-44 | 87.9 (85.0 to 90.3) | -2.76 (-6.43, 0.92) | 0.140 | 0.97 (0.93, 1.01) | 0.140 |
| 45-54 | 80.5 (76.8 to 83.7) | -10.11 (-14.46, -5.75) | <0.0001 | 0.89 (0.84, 0.94) | <0.0001 |
| 55-70 | 77.0 (73.2 to 80.4) | -12.33 (-17.12, -7.55) | <0.0001 | 0.86 (0.82, 0.92) | <0.0001 |
| **Sex** |  |  |  |  |  |
| Male | 84.4 (81.9 to 86.6) | 0 (ref) |  | 1 (ref) |  |
| Female | 85.3 (82.8 to 87.5) | -0.96 (-4.18, 2.26) | 0.56 | 0.99 (0.95, 1.03) | 0.56 |
| **Education** |  |  |  |  |  |
| No schooling | 83.0 (78.1 to 86.9) | 0 (ref) |  | 1 (ref) |  |
| Primary | 83.9 (81.3 to 86.3) | -1.13 (-5.71, 3.45) | 0.63 | 0.99 (0.93, 1.04) | 0.63 |
| Secondary or higher | 86.7 (84.4 to 88.7) | 0.42 (-5.01, 5.85) | 0.88 | 1.00 (0.94, 1.07) | 0.88 |

Bangladesh, Myanmar, Nepal, Sir Lanka and Timor-Leste have a body mass index target of less than 23 kg/m^2^.

Lifestyle targets

|  | Prevalence (%) | Estimated average marginal effect (95% CI) | *P* values | Risk ratio (95% CI) | *P* values |
| --- | --- | --- | --- | --- | --- |
| **Not currently smoking** | | | | | |
| **Age, years** |  |  |  |  |  |
| 18-34 | 85.5 (72.1 to 88.4) | 0 (ref) |  | 1 (ref) |  |
| 35-44 | 81.4 (77.6 to 84.6) | -1.97 (-6.23, 2.29) | 0.37 | 0.99 (0.97, 1.01) | 0.370 |
| 45-54 | 83.6 (80.6 to 86.3) | 1.15 (-2.87, 5.17) | 0.57 | 1.01 (0.98, 1.03) | 0.580 |
| 55-69 | 81.6 (78.5 to 82.3) | 2.71 (-1.52, 6.94) | 0.21 | 1.01 (0.99, 1.04) | 0.210 |
| **Sex** |  |  |  |  |  |
| Male | 66.1 (62.9 to 69.2) | 0 (ref) |  | 1 (ref) |  |
| Female | 96.8 (95.8 to 97.5) | 33.44 (30.35, 36.53) | <0.0001 | 1.20 (1.18, 1.23) | <0.0001 |
| **Education** |  |  |  |  |  |
| No schooling | 86.0 (82.7 to 88.8) | 0 (ref) |  | 1 (ref) |  |
| Primary | 80.4 (77.6 to 82.9) | 3.15 (-0.80, 7.10) | 0.12 | 1.02 (1.00, 1.04) | 0.12 |
| Secondary or higher | 83.8 (81.2 to 86.2) | 11.81 (6.95, 16.68) | <0.0001 | 1.07 (1.04, 1.10) | <0.0001 |
| **Not currently drinking** | | | | | |
| **Age, years** |  |  |  |  |  |
| 18-34 | 82.8 (79.1 to 85.9) | 0 (ref) |  | 1 (ref) |  |
| 35-44 | 80.4 (76.1 to 84.0) | -3.96 (-8.41, 0.49) | 0.08 | 0.98 (0.95, 1.00) | 0.08 |
| 45-54 | 83.2 (79.8 to 86.1) | 0.00 (-4.17, 4.18) | 1.00 | 1.00 (0.98, 1.02) | 1.00 |
| 55-70 | 85.6 (83.0 to 87.8) | 1.97 (-1.99, 5.93) | 0.33 | 1.01 (0.99, 1.03) | 0.33 |
| **Sex** |  |  |  |  |  |
| Male | 72.7 (69.2 to 75.9) | 0 (ref) |  | 1 (ref) |  |
| Female | 91.5 (89.9 to 92.8) | 20.92 (17.75, 24.09) | <0.0001 | 1.12 (1.10, 1.14) | <0.0001 |
| **Education** |  |  |  |  |  |
| No schooling | 87.3 (83.4 to 90.4) | 0 (ref) |  | 1 (ref) |  |
| Primary | 85.9 (83.3 to 88.2) | 4.44 (0.95, 7.94) | 0.01 | 1.02 (1.01, 1.04) | 0.01 |
| Secondary or higher | 77.0 (73.9 to 79.9) | 2.72 (-1.43, 6.87) | 0.20 | 1.02 (0.99, 1.04) | 0.20 |
| **Sufficient physical activity** | | | | | |
| **Age, years** |  |  |  |  |  |
| 18-34 | 76.8 (72.6 to 80.6) | 0 (ref) |  | 1 (ref) |  |
| 35-44 | 76.0 (72.1 to 79.4) | 2.54 (-2.50, 7.58) | 0.32 | 1.04 (0.97, 1.11) | 0.33 |
| 45-54 | 66.8 (62.8 to 70.6) | -2.38 (-8.08, 3.32) | 0.41 | 0.97 (0.89, 1.05) | 0.41 |
| 55-70 | 43.7 (40.0 to 47.5) | -23.13 (-28.88, -17.39) | <0.0001 | 0.67 (0.61, 0.75) | <0.0001 |
| **Sex** |  |  |  |  |  |
| Male | 65.0 (61.8 to 68.1) | 0 (ref) |  | 1 (ref) |  |
| Female | 65.8 (62.7 to 68.8) | -2.96 (-6.78, 0.86) | 0.13 | 0.96 (0.90, 1.01) | 0.13 |
| **Education** |  |  |  |  |  |
| No schooling | 69.6 (64.9 to 74.0) | 0 (ref) |  | 1 (ref) |  |
| Primary | 65.3 (61.7 to 68.8) | -2.62 (-7.46, 2.23) | 0.29 | 0.96 (0.89, 1.03) | 0.29 |
| Secondary or higher | 62.6 (59.1 to 65.9) | -3.82 (-10.36, 2.72) | 0.25 | 0.94 (0.85, 1.04) | 0.25 |
| **Dietary fruit and vegetables ≥5 servings/day** | | | | | |
| **Age, years** |  |  |  |  |  |
| 18-34 | 13.9 (10.9 to 17.6) | 0 (ref) |  | 1 (ref) |  |
| 35-44 | 14.4 (11.5 to 17.7) | 0.26 (-4.06, 4.58) | 0.91 | 1.02 (0.78, 1.33) | 0.91 |
| 45-54 | 15.8 (13.1 to 18.9) | -1.22 (-5.94, 3.50) | 0.61 | 0.92 (0.68, 1.25) | 0.61 |
| 55-70 | 20.4 (17.6 to 23.5) | 1.53 (-2.98, 6.04) | 0.51 | 1.10 (0.83, 1.44) | 0.51 |
| **Sex** |  |  |  |  |  |
| Male | 17.2 (14.7 to 20.1) | 0 (ref) |  | 1 (ref) |  |
| Female | 15.4 (13.4 to 17.6) | -0.29 (-3.30, 2.72) | 0.85 | 0.98 (0.82, 1.18) | 0.85 |
| **Education** |  |  |  |  |  |
| No schooling | 5.2 (3.9 to 7.1) | 0 (ref) |  | 1 (ref) |  |
| Primary | 13.3 (11.1 to 16.0) | 5.40 (1.92, 8.87) | <0.0001 | 1.64 (1.15, 2.32) | 0.01 |
| Secondary or higher | 25.8 (22.7 to 29.1) | 11.81 (7.67, 15.94) | <0.0001 | 2.39 (1.66, 3.45) | <0.0001 |

**Appendix 6: Sensitivity analysis**

Supplementary Table S8: Prevalence of treatment, metabolic and lifestyle targets for secondary prevention of CVD

|  | Prevalence (95% CI) | | | |
| --- | --- | --- | --- | --- |
|  | Overall | Upper-Middle Income Countries | Lower-Middle Income Countries | Low-Income Countries |
| 1. **Treatment targets** | | | | |
| Antihypertensive drug | 26.57 (24.86, 28.35) | 35.42 (31.13, 39.97) | 24.83 (22.55, 27.25) | 16.43 (13.53, 19.82) |
| Statins | 12.60 (11.36, 13.95) | 19.42 (16.17, 23.14) | 9.69 (8.42, 11.13) | 10.28 (7.11, 14.65) |
| Aspirin | 23.35 (21.74, 25.05) | 36.78 (31.71, 42.15) | 17.96 (16.19, 19.88) | 17.61 (14.23, 21.60) |
| 1. **Metabolic targets** | | | | |
| Body mass index <25 kg/m² | 43.99 (41.15, 46.88) | 26.37 (21.80, 31.52) | 47.25 (44.39, 50.13) | 64.91 (60.61, 68.98) |
| Blood pressure <130/80 mm Hg | 35.34 (33.19, 37.56) | 33.13 (27.97, 38.73) | 36.84 (34.28, 39.48) | 34.14 (30.72, 37.75) |
| Total cholesterol <4.0 mmol/L | 44.50 (42.34, 46.68) | 40.64 (36.76, 44.63) | 42.19 (39.36, 45.07) | 59.67 (55.31, 63.88) |
| Fasting blood glucose <6.1 mmol/L | 82.01 (80.31, 83.60) | 78.10 (74.43, 81.38) | 82.08 (79.85, 84.11) | 88.96 (85.87, 91.45) |
| 1. **Lifestyle targets** | | | | |
| Not currently smoking | 79.84 (77.46, 82.02) | 71.80 (66.05, 76.92) | 81.39 (79.37, 83.26) | 89.14 (86.39, 91.39) |
| Not currently drinking | 76.60 (74.69, 78.41) | 79.63 (74.85, 83.71) | 75.46 (72.90, 77.86) | 75.04 (71.05, 78.64) |
| Sufficient physical activity | 62.23 (60.10, 64.31) | 50.71 (44.90, 56.50) | 63.49 (60.76, 66.14) | 78.93 (76.02, 81.57) |
| Dietary fruit and vegetable ≥5 servings/day | 16.82 (15.32, 18.43) | 18.40 (14.97, 22.41) | 18.52 (16.59, 20.61) | 7.97 (6.19, 10.21) |

Prevalence was calculated using adjusted weight, giving each country equal weight. Bangladesh, Myanmar, Nepal, Sir Lanka and Timor-Leste have a body mass index target of less than 23 kg/m^2^.

Supplementary Table S9: Prevalence of treatment, metabolic and lifestyle targets for secondary prevention of CVD, by region

Treatment targets

| Region | Prevalence (95% CI) | | | |
| --- | --- | --- | --- | --- |
|  | Antihypertensive drug | Aspirin | Statins | All 3 targets met |
| Africa | 13.64 (11.18, 16.55) | 11.52 (9.47, 13.93) | 6.56 (4.82, 8.87) | 2.87 (1.78, 4.58) |
| Americas | 24.53 (18.50, 31.77) | 19.60 (14.43, 26.07) | 8.26 (5.47, 12.27) | 2.79 (1.56, 4.94) |
| Western Pacific | 14.80 (12.38, 17.60) | 17.07 (13.74, 20.99) | 6.25 (4.96, 7.86) | 2.41 (1.56, 3.70) |
| European | 43.99 (41.09, 46.93) | 34.40 (31.51, 37.41) | 12.66 (10.73, 14.88) | 7.34 (5.95, 9.02) |
| Eastern Mediterranean | 39.14 (33.56, 45.01) | 44.01 (38.19, 50.01) | 27.56 (22.32, 33.49) | 17.82 (13.77, 22.75) |
| South-East Asia | 26.99 (21.81, 32.87) | 15.96 (12.90, 19.60) | 17.19 (13.86, 21.12) | 6.62 (4.96, 8.79) |

Prevalence was calculated using adjusted weight, giving each country equal weight.

Metabolic targets

| Region | Prevalence (95% CI) | | | | |
| --- | --- | --- | --- | --- | --- |
|  | Body mass index <25 kg/m² | Blood pressure <130/80 mm Hg | Total cholesterol <4.0 mmol/L | Fasting blood-glucose <6.1 mmol/L | All 4 targets met |
| Africa | 64.75 (61.17, 68.18) | 42.50 (39.19, 45.89) | 58.86 (55.15, 62.47) | 88.38 (85.42, 90.81) | 22.32 (19.56, 25.35) |
| Americas | 37.11 (29.45, 45.47) | 54.63 (46.52, 62.51) | 26.33 (20.68, 32.89) | 78.01 (70.86, 83.81) | 7.70 (5.21, 11.23) |
| Western Pacific | 31.80 (23.68, 41.21) | 43.45 (36.42, 50.75) | 36.18 (29.99, 42.87) | 79.44 (73.43, 84.39) | 7.36 (5.11, 10.50) |
| European | 26.90 (24.06, 29.95) | 17.07 (14.96, 19.40) | 32.53 (29.63, 35.58) | 80.28 (77.90, 82.46) | 3.87 (2.87, 5.21) |
| Eastern Mediterranean | 38.80 (32.77, 45.20) | 30.91 (25.68, 36.68) | 48.42 (42.55, 54.33) | 75.64 (70.64, 80.03) | 10.54 (7.87, 13.97) |
| South-East Asia | 49.28 (42.78, 55.81) | 36.72 (30.98, 42.86) | 44.59 (37.98, 51.40) | 83.12 (78.27, 87.07) | 13.84 (9.17, 20.36) |

Prevalence was calculated using adjusted weight, giving each country equal weight. Bangladesh, Myanmar, Nepal, Sir Lanka and Timor-Leste have a body mass index target of less than 23 kg/m^2^.

Lifestyle targets

| Region | Prevalence (95% CI) | | | | |
| --- | --- | --- | --- | --- | --- |
|  | Not currently smoking | Not currently drinking | Sufficient physical activity | Fruit and vegetables ≥5 servings/day | All 4 targets met |
| Africa | 88.60 (86.01, 90.76) | 70.41 (66.28, 74.23) | 74.10 (70.69, 77.24) | 11.91 (9.53, 14.78) | 5.17 (3.97, 6.70) |
| Americas | 85.11 (78.95, 89.71) | 60.34 (52.49, 67.69) | 54.95 (47.25, 62.43) | 4.55 (2.58, 7.93) | 0.65 (0.20, 2.09) |
| Western Pacific | 61.41 (54.65, 67.76) | 72.07 (66.44, 77.08) | 65.74 (58.67, 72.17) | 13.23 (9.56, 18.04) | 4.54 (2.98, 6.86) |
| European | 81.58 (79.10, 83.83) | 70.43 (67.51, 73.18) | 54.23 (51.01, 57.41) | 31.59 (28.68, 34.65) | 12.67 (10.90, 14.69) |
| Eastern Mediterranean | 78.52 (72.49, 83.53) | 96.70 (91.31, 98.79) | 46.09 (40.33, 51.96) | 16.68 (12.84, 21.39) | 5.12 (3.14, 8.27) |
| South-East Asia | 79.38 (74.89, 83.24) | 87.96 (84.08, 90.99) | 66.99 (61.52, 72.03) | 13.36 (10.11, 17.45) | 5.36 (3.92, 7.28) |

Prevalence was calculated using adjusted weight, giving each country equal weight.

Supplementary Table S10: Prevalence of guideline-recommended targets for all components of treatment, metabolic and lifestyle in patients with CVD by World Bank income group and region

By World Bank income group

|  | Prevalence (95% CI) | | | |
| --- | --- | --- | --- | --- |
|  | Overall | Upper-Middle Income Countries | Lower-Middle Income Countries | Low-Income Countries |
| Treatment targets all met | 6.59 (5.72, 7.57) | 12.60 (10.15, 15.54) | 3.82 (3.04, 4.78) | 5.26 (3.16, 8.63) |
| Metabolic targets all met | 12.33 (10.95, 13.86) | 6.98 (5.33, 9.09) | 12.38 (10.53, 14.51) | 21.96 (19.04, 25.18) |
| Lifestyle targets all met | 6.43 (5.65, 7.31) | 6.45 (4.98, 8.32) | 7.07 (6.01, 8.29) | 4.16 (3.00, 5.76) |

Prevalence was calculated using adjusted weight, giving each country equal weight.

By region

|  | Prevalence (95% CI) | | | | | |
| --- | --- | --- | --- | --- | --- | --- |
|  | Africa | Americas | Western Pacific | Europe | Eastern Mediterranean | South-East Asia |
| Treatment targets all met | 2.87 (1.78, 4.58) | 2.79 (1.56, 4.94) | 2.41 (1.56, 3.70) | 7.34 (5.95, 9.02) | 17.82 (13.77, 22.75) | 6.62 (4.96, 8.79) |
| Metabolic targets all met | 22.32 (19.56, 25.35) | 7.70 (5.21, 11.23) | 7.36 (5.11, 10.50) | 3.87 (2.87, 5.21) | 10.54 (7.87, 13.97) | 13.84 (9.17, 20.36) |
| Lifestyle targets all met | 5.17 (3.97, 6.70) | 0.65 (0.20, 2.09) | 4.54 (2.98, 6.86) | 12.67 (10.90, 14.69) | 5.12 (3.14, 8.27) | 5.36 (3.92, 7.28) |

Prevalence was calculated using adjusted weight, giving each country equal weight.

Supplementary Table S11: Prevalence of treatment, metabolic and lifestyle targets for secondary prevention of CVD (over 40 years), by income group and region

|  | Prevalence (95% CI) | | | |
| --- | --- | --- | --- | --- |
|  | Overall | Upper-Middle Income Countries | Lower-Middle Income Countries | Low-Income Countries |
| **Treatment targets** | | | | |
| Antihypertensive drug | 33.95 (31.13, 36.89) | 46.11 (40.54, 51.79) | 32.01 (28.54, 35.69) | 24.26 (17.44, 32.70) |
| Statins | 18.87 (16.12, 21.97) | 25.70 (21.04, 30.98) | 17.32 (13.73, 21.61) | 14.59 (8.58, 23.72) |
| Aspirin | 26.89 (23.98, 30.02) | 43.80 (38.17, 49.60) | 21.92 (18.41, 25.90) | 19.09 (12.40, 28.23) |
| All 3 targets met | 10.80 (8.63, 13.44) | 17.23 (13.36, 21.93) | 7.76 (5.25, 11.34) | 10.70 (5.47, 19.87) |

Sample weights are re-adjusted based on each country’s 2019 population aged 40-69.

By region

| Region | Prevalence (95% CI) | | | |
| --- | --- | --- | --- | --- |
|  | Antihypertensive drug | Aspirin | Statins | All 3 targets met |
| Africa | 22.41 (18.17, 27.31) | 19.38 (15.18, 24.41) | 12.36 (8.88, 16.94) | 7.83 (5.12, 11.80) |
| Americas | 30.04 (23.56, 37.42) | 20.51 (15.00, 27.38) | 13.29 (8.90, 19.38) | 5.62 (3.07, 10.05) |
| Western Pacific | 39.56 (35.05, 44.26) | 33.41 (29.39, 37.69) | 8.01 (6.09, 10.47) | 3.72 (2.46, 5.57) |
| European | 54.29 (51.43, 57.11) | 36.99 (34.00, 40.09) | 13.84 (11.75, 16.23) | 8.74 (7.10, 10.71) |
| Eastern Mediterranean | 44.36 (35.84, 53.22) | 37.86 (28.91, 47.72) | 26.54 (19.29, 35.32) | 17.40 (11.50, 25.44) |
| South-East Asia | 31.65 (26.92, 36.78) | 23.66 (18.67, 29.50) | 21.51 (16.25, 27.91) | 10.03 (6.33, 15.54) |

Sample weights are re-adjusted based on each country’s 2019 population aged 40-69.

Supplementary Table S12: Prevalence of blood pressure <140/90 mm Hg, by income group and region

|  | Prevalence (95% CI) | | | |
| --- | --- | --- | --- | --- |
|  | Overall | Upper-Middle Income Countries | Lower-Middle Income Countries | Low-Income Countries |
| Blood pressure <140/90 mm Hg | 67.44 (65.40, 69.42) | 56.88 (52.71, 60.96) | 69.36 (66.63, 71.97) | 70.32 (65.94, 74.37) |
| All metabolic target met | 35.44 (33.09, 37.87) | 15.78 (12.77, 19.35) | 34.48 (31.54, 37.54) | 55.23 (50.34, 60.02) |

Sample weights are re-adjusted based on each country’s 2019 population aged 18-69.

By region

|  | Prevalence (95% CI) | | | | | |  |
| --- | --- | --- | --- | --- | --- | --- | --- |
|  | Africa | Americas | Western Pacific | Europe | Eastern Mediterranean | South-East Asia | |
| Blood pressure <130/80 mm Hg | 73.20 (70.02, 76.16) | 81.06 (76.26, 85.08) | 72.88 (66.65, 78.31) | 43.49 (40.07, 46.97) | 59.02 (53.70, 64.14) | 69.89 (65.69, 73.78) | |
| All metabolic target met | 52.05 (48.07, 56.01) | 21.62 (17.00, 27.09) | 44.10 (36.10, 52.42) | 13.85 (11.35, 16.80) | 25.71 (20.41, 31.84) | 28.84 (25.08, 32.93) | |

Sample weights are re-adjusted based on each country’s 2019 population aged 18-69.

Supplementary Table S13: Prevalence of total cholesterol <5.0 mmol/L (190 mg/mL), by income group and region

|  | Prevalence (95% CI) | | | |
| --- | --- | --- | --- | --- |
|  | Overall | Upper-Middle Income Countries | Lower-Middle Income Countries | Low-Income Countries |
| Total cholesterol <5.0 mmol/L | 75.49 (73.28, 77.57) | 65.74 (61.90, 69.39) | 73.58 (70.56, 76.40) | 89.81 (86.38, 92.46) |
| All metabolic target met | 35.44 (33.09, 37.87) | 15.78 (12.77, 19.35) | 34.48 (31.54, 37.54) | 55.23 (50.34, 60.02) |

Sample weights are re-adjusted based on each country’s 2019 population aged 18-69.

By region

|  | Prevalence (95% CI) | | | | | |
| --- | --- | --- | --- | --- | --- | --- |
|  | Africa | Americas | Western Pacific | Europe | Eastern Mediterranean | South-East Asia |
| Total cholesterol <5.0 mmol/L | 89.96 (87.78, 91.79) | 67.20 (61.27, 72.63) | 62.64 (54.59, 70.05) | 65.47 (62.55, 68.27) | 74.42 (68.99, 79.19) | 71.48 (67.00, 75.58) |
| All metabolic target met | 52.05 (48.07, 56.01) | 21.62 (17.00, 27.09) | 44.10 (36.10, 52.42) | 13.85 (11.35, 16.80) | 25.71 (20.41, 31.84) | 28.84 (25.08, 32.93) |

Sample weights are re-adjusted based on each country’s 2019 population aged 18-69.

**Appendix 7: STROBE Checklist**

Checklist of items that should be included in reports of cross-sectional studies

|  | Item No | Recommendation | Item  No |
| --- | --- | --- | --- |
| **Title and abstract** | 1 | (*a*) Indicate the study’s design with a commonly used term in the title or the abstract | 1 |
|  |  | (*b*) Provide in the abstract an informative and balanced summary of what was done and what was found | 1, 2 |
| Introduction | | |  |
| Background/rationale | 2 | Explain the scientific background and rationale for the investigation being reported | 2 |
| Objectives | 3 | State specific objectives, including any prespecified hypotheses | 2 |
| Methods | | |  |
| Study design | 4 | Present key elements of study design early in the paper | 2, 3 |
| Setting | 5 | Describe the setting, locations, and relevant dates, including periods of recruitment, exposure, follow-up, and data collection | 2, 3 |
| Participants | 6 | (*a*) Give the eligibility criteria, and the sources and methods of selection of participants | 2, 3 |
| Variables | 7 | Clearly define all outcomes, exposures, predictors, potential confounders, and effect modifiers. Give diagnostic criteria, if applicable | 3, 4 |
| Data sources/ measurement | 8* | For each variable of interest, give sources of data and details of methods of assessment (measurement). Describe comparability of assessment methods if there is more than one group | 3, 4 |
| Bias | 9 | Describe any efforts to address potential sources of bias | 7, 8 |
| Study size | 10 | Explain how the study size was arrived at | 4 |
| Quantitative variables | 11 | Explain how quantitative variables were handled in the analyses. If applicable, describe which groupings were chosen and why | 3, 4 |
| Statistical methods | 12 | (*a*) Describe all statistical methods, including those used to control for confounding | 4 |
|  |  | (*b*) Describe any methods used to examine subgroups and interactions | 4 |
|  |  | (*c*) Explain how missing data were addressed | 4 |
|  |  | (*d*) If applicable, describe analytical methods taking account of sampling strategy | 4 |
|  |  | (*e*) Describe any sensitivity analyses | 4 |
| Results | | |  |
| Participants | 13* | (a) Report numbers of individuals at each stage of study—eg numbers potentially eligible, examined for eligibility, confirmed eligible, included in the study, completing follow-up, and analysed | 4 |
|  |  | (b) Give reasons for non-participation at each stage | 4 |
|  |  | (c) Consider use of a flow diagram | 4 |
| Descriptive data | 14* | (a) Give characteristics of study participants (eg demographic, clinical, social) and information on exposures and potential confounders | 4, 8, 9 |
|  |  | (b) Indicate number of participants with missing data for each variable of interest | 4 |
| Outcome data | 15* | Report numbers of outcome events or summary measures | 4 |
| Main results | 16 | (*a*) Give unadjusted estimates and, if applicable, confounder-adjusted estimates and their precision (eg, 95% confidence interval). Make clear which confounders were adjusted for and why they were included | 4, 5 |
|  |  | (*b*) Report category boundaries when continuous variables were categorized | 4, 5 |
|  |  | (*c*) If relevant, consider translating estimates of relative risk into absolute risk for a meaningful time period | 4, 5 |
| Other analyses | 17 | Report other analyses done—eg analyses of subgroups and interactions, and sensitivity analyses | 5, 6 |
| Discussion | | |  |
| Key results | 18 | Summarise key results with reference to study objectives | 6 |
| Limitations | 19 | Discuss limitations of the study, taking into account sources of potential bias or imprecision. Discuss both direction and magnitude of any potential bias | 7, 8 |
| Interpretation | 20 | Give a cautious overall interpretation of results considering objectives, limitations, multiplicity of analyses, results from similar studies, and other relevant evidence | 6, 7 |
| Generalisability | 21 | Discuss the generalisability (external validity) of the study results | 8 |
| Other information | | |  |
| Funding | 22 | Give the source of funding and the role of the funders for the present study and, if applicable, for the original study on which the present article is based |  |

*Give information separately for exposed and unexposed groups.
